# A Multi-UAV Cooperative Path-Planning Method for Complex Obstacle Environments

**DOI:** 10.3390/s26165242

**Published:** 2026-08-19

**Authors:** Long Wen, Hui Tan, Yuxin Liu, Xinyang Zhao, Shaowang Xie, Bo Zhao

**Affiliations:** 1 Power Engineering College, Naval University of Engineering, No. 717 Jiefang Road, Wuhan 430033, China; 2 School of Mechanical Engineering and Electronic Information, China University of Geosciences, No. 388 Lumo Road, Wuhan 430074, China; 3 National Key Laboratory of Electromagnetic Effect and Security on Marine Equipment, China Ship Development and Design Center (CSDDC), Wuhan 430061, China

**Keywords:** multi-UAV, collaborative path planning, MARL, POMDP

## Abstract

Reinforcement learning techniques have been widely applied to multi-UAV cooperative path-planning tasks. However, existing multi-agent reinforcement learning methods are still affected by environmental non-stationarity, cooperation difficulties among agents, and low utilization efficiency of experience samples in complex obstacle environments. These issues often lead to slow convergence and unstable training performance. To address these problems, an Improved Experience Replay Multi-Agent Deep Deterministic Policy Gradient (IER-MADDPG) algorithm is proposed for multi-UAV cooperative path planning. First, a cooperative path-planning model is established under the Centralized Training Distributed Execution framework. Second, a dual-layer replay buffer structure consisting of a global replay buffer and a local replay buffer is designed to preserve both global cooperative information and individual experience. Third, a fusion experience sampling mechanism is introduced by combining prioritized experience replay and random uniform sampling to improve sample utilization efficiency and training stability. Finally, training experiments were conducted in environments with different obstacle configurations to evaluate the proposed method. Experimental results demonstrate that IER-MADDPG outperforms other comparison algorithms in terms of convergence speed, training stability, and path-planning performance.

## 1. Introduction

In recent years, cooperative operations of unmanned aerial vehicles (UAVs) have shown broad application prospects in maritime search [1], environmental inspection [2] and emergency rescue [3]. As the demand for low-altitude operations becomes more diverse and complex, a single UAV exhibits limitations in task efficiency, environmental coverage, and system robustness, making it difficult to satisfy the requirements of large-scale missions in complex scenarios [4]. Consequently, multi-UAV cooperative operation has become an important research direction in intelligent unmanned systems [5]. However, obstacle distribution in complex environments and coupling constraints among UAVs have a significant impact on cooperative path-planning performance [6]. These factors make efficient cooperative path planning a challenging task.

With the development of reinforcement learning, cooperative path-planning methods based on the partially observable Markov decision process (POMDP) have become an important research direction in the field of autonomous decision making for multiple UAVs [7]. These methods establish the mapping relationships among environmental states, agent actions, and rewards, which enable autonomous cooperative decision making in complex dynamic environments [8]. Zhang et al. [9] formulated the multi-UAV cooperative path-planning problem as a POMDP and proposed a PE-MADDPG-based method. By integrating prioritized experience replay, adaptive exploration noise, and an attention mechanism, the method improves decision-making efficiency and training stability in complex dynamic environments. Liu et al. [10] formulated the UAV swarm path-planning problem as a POMDP and developed a cooperative decision-making method for continuous action spaces based on MADDPG. This method addresses the challenges of partial observability and complex constraints in UAV swarm cooperative path planning under dynamic environments. These studies demonstrate the effectiveness of POMDP and multi-agent reinforcement learning methods in multi-UAV cooperative path planning. However, challenges related to sample utilization, training stability, and cooperation efficiency still remain in complex environments. Therefore, developing efficient cooperative path-planning methods remains an important research topic.

MADDPG can effectively improve continuous decision-making capability and cooperative control performance in multi-UAV cooperative path-planning tasks. Liu et al. [11] proposed a dual time scale hierarchical MADDPG method. The method establishes a hierarchical cooperative decision-making framework, which addresses the problems of high decision dimensionality, slow training convergence, and insufficient cooperation efficiency in multi-UAV cooperative search tasks. Liu et al. [12] proposed an AMCPP method that combines a rotational artificial potential field with an improved multi-agent deep deterministic policy gradient algorithm. The proposed method introduces a rotational potential field guidance mechanism and a cooperative reinforcement learning framework, which addresses the problems that traditional MADDPG tends to fall into local optima and exhibits limited cooperative obstacle avoidance capability in complex dynamic-obstacle environments. Existing studies indicate that MADDPG and its improved variants provide effective solutions for multi-UAV cooperative path planning and exhibit strong cooperative decision-making capability in complex environments. However, existing methods still suffer from insufficient sample utilization, poor training stability, and limited global cooperation capability in environments with dense obstacles. Therefore, the development of efficient multi-UAV cooperative path-planning methods for complex dynamic environments remains a challenging task with significant research importance and engineering value.

To address the challenges discussed above, this paper proposes an Improved Experience Replay MADDPG-based multi-UAV cooperative path-planning algorithm, which is referred to as IER-MADDPG. Compared with the conventional MADDPG framework, which typically employs a single replay buffer with uniform random sampling, IER-MADDPG introduces a dual-layer replay structure and a fused experience sampling mechanism. The global replay buffer preserves joint interaction experiences among multiple UAVs, while the local replay buffer emphasizes recent task experiences with high learning value. Prioritized sampling and random sampling are combined to improve the utilization of important experiences while maintaining sample diversity. Integrated with the centralized training and decentralized execution framework, this design enables each UAV to generate actions independently from local observations, while the Critic networks perform centralized value evaluation using joint observations and joint actions. Therefore, IER-MADDPG provides a task-oriented experience organization and sharing mechanism for multi-UAV cooperative policy learning, contributing to improved sample utilization efficiency and training stability. The main contributions of this paper are summarized as follows.

(1) A multi-UAV cooperative path-planning simulation environment is developed by considering UAV kinematics, cooperative constraints, and complex obstacle environments.

(2) The multi-UAV cooperative path-planning problem is formulated as a POMDP to achieve cooperative autonomous decision making in complex dynamic environments.

(3) An IER-MADDPG algorithm is proposed by incorporating experience replay and experience sharing mechanisms to improve sample utilization and training stability.

(4) Extensive simulation experiments were conducted to evaluate the effectiveness and robustness of the proposed method under different obstacle densities and UAV scales.

The remainder of this paper is organized as follows. Section 2 reviews related work on multi-UAV cooperative path planning. Section 3 presents the problem definition and the UAV kinematic model. Section 4 introduces the proposed IER-MADDPG method. Section 5 presents the experimental results and analysis. Finally, Section 6 concludes the paper.

## 2. Related Works

Multi-UAV cooperative path planning is a fundamental technology in fields such as low-altitude security [13] and border patrol [14]. Traditional path-planning methods are mainly based on rule-based mechanisms, heuristic search algorithms, or geometric models. These methods often suffer from limited environmental adaptability, insufficient real-time decision-making capability, and low cooperation efficiency in complex dynamic environments [15]. Aslan et al. [16] proposed a geometric path-planning algorithm called BaF, which is based on a greedy heuristic strategy. The algorithm employs a bidirectional path generation mechanism and a path combination mechanism, which enable rapid path search and optimization in complex environments. As a result, path-planning efficiency and flight safety are improved. Lin et al. [17] introduced causal representation learning into multi-UAV collision avoidance. The method improves robustness and generalization capability in dynamic and unseen environments. Ding et al. [18] proposed a perception-aware cooperative path-planning method for multi-UAV systems in urban wind fields. The method models wind speed, obstacle distance, and inter-UAV states as environmental perception information and introduces an enhanced D3QN algorithm to improve path-planning robustness under complex urban conditions.

In recent years, UAV path-planning methods based on POMDP and deep reinforcement learning have become an important research direction for autonomous decision making in complex dynamic environments. Wang et al. [19] proposed a joint trajectory planning method that combines federated multi-agent reinforcement learning with an artificial potential field. The method formulates the multi-UAV trajectory planning problem as POMDP and achieves cooperative trajectory optimization and task offloading decision making in complex dynamic environments. Chun et al. [20] proposed a deep reinforcement learning-based path-planning model, which is referred to as PPM. The method establishes a Markov decision process model and incorporates feature engineering and a backtracking mechanism, which address the feasibility and optimization problems of UAV path planning under localization errors. Shu et al. [21] proposed an energy-saving multi-agent deep reinforcement learning algorithm for the drone routing problem. The method integrates a drone energy consumption model into multi-agent deep reinforcement learning, which improves routing performance and energy efficiency compared with traditional heuristic-based methods. Liao et al. [22] investigated the multi-UAV target search problem in environments with static obstacles and dynamic escape targets. They modeled the task as a decentralized partially observable Markov decision process and proposed an ETS-MAPPO algorithm, which improves target search performance and area coverage in complex environments. Jiang et al. [23] proposed a three-dimensional UAV path-planning method based on an improved teaching–learning optimization algorithm. This method improves global search capability, convergence speed, and path quality.

Gao et al. [24] proposed a scalable path-planning algorithm based on MADDPG. The algorithm constructs a neighborhood observation space and a hybrid experience sampling mechanism, which improve training stability and cooperative decision-making efficiency in multi-agent path-planning tasks. Cai et al. [25] proposed an EA-MADDPG cooperative decision-making algorithm. The algorithm introduces a balanced reward mechanism and an action enhancement strategy, which improve cooperative control capability and target interception performance in complex three-dimensional environments. Zhang et al. [26] proposed a multi-UAV game decision making method that combines particle swarm optimization with M3DDPG. The method optimizes the experience sampling process and the cooperative training mechanism, which alleviate the problems of sparse samples and local optima during reinforcement learning training. Zhou et al. [27] proposed an anti-interference path-planning method based on the PDS-DDPG algorithm and the TOPEM mechanism. The method establishes a post-decision-state model and an interruption probability experience memory mechanism, which improve robustness and anti-interference capability in complex communication environments. Li et al. [28] proposed a global path-planning method based on HPER-MADDPG. The method combines a hindsight-prioritized experience replay mechanism with a multi-agent reinforcement learning framework, which improve training efficiency and path-planning performance in environments with complex constraints. Fan et al. [29] proposed a multi-agent deep reinforcement learning-based trajectory planning method for heterogeneous task requirements. The method employs personalized policy updates and diversified experience sampling, which achieve cooperative trajectory optimization for multiple UAVs and improve task delay and task completion performance. Zhang and Wen et al. [30] proposed the MA-JTATO joint optimization framework. The framework employs MADDPG for multi-UAV cooperative trajectory planning and integrates task association with resource allocation, which improve the overall performance of UAV-assisted edge computing systems. However, stable and efficient multi-UAV cooperative path planning remains a challenging problem in complex environments that contain obstacle constraints and dynamic uncertainties.

## 3. Problem Definition

This section presents the task description of UAV-based target search and the definition of the UAV model. These contents provide the foundation for the design of the proposed algorithm and the simulation experiments.

### 3.1. Task Description of Multi-UAV Collaborative Path Planning

**Figure 1:**
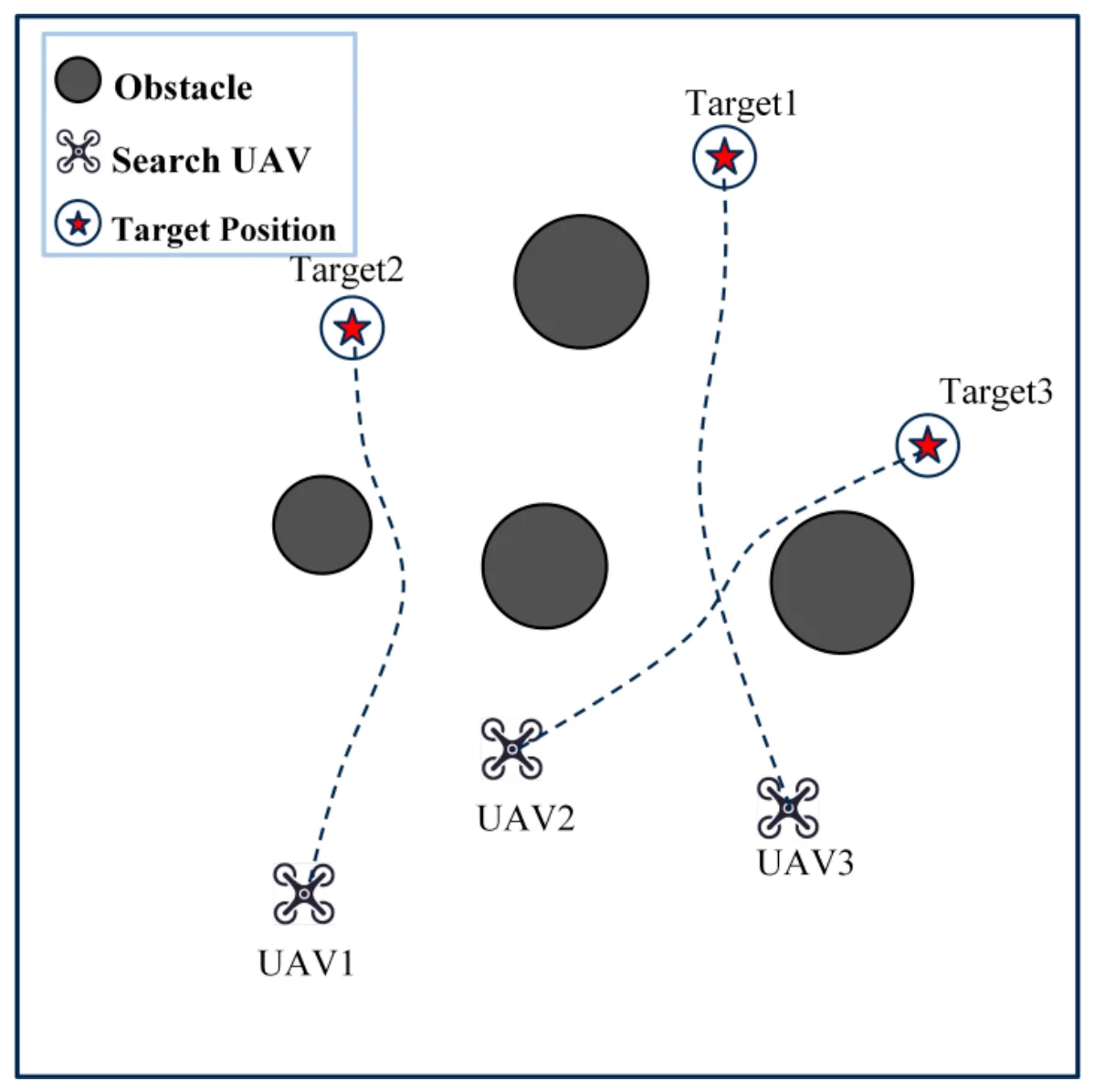
Schematic diagram of multi-UAV cooperative search task scenario.

The MARL algorithm is employed to control multiple UAVs, which perform cooperative path-planning tasks in a complex environment that contains obstacles. The task scenario is defined in a bounded two-dimensional space, which contains *N* (N≥3) homogeneous UAVs, and a predefined target position. Each UAV perceives obstacle information from its surrounding environment and adjusts its control actions to avoid hazardous areas, such as obstacles, and reach the target position safely. All UAVs that participate in the path-planning task share the same kinematic model. At time t, after the UAV swarm executes a joint action, each UAV transitions to a new state, receives feedback from the environment, and updates its policy. The multi-UAV cooperative path-planning task consists of the following objectives. The cooperative algorithm guides all UAVs to reach the predefined target position within a specified time through optimal paths. Collisions between UAVs and obstacles, as well as collisions among UAVs, should be avoided during the path-planning process. Each UAV should remain within the boundaries of the planning area. As shown in Figure 1, multiple UAVs cooperate to generate optimal paths and reach the destination collectively, thereby accomplishing the cooperative path-planning task. The dashed line indicates the flight path of the UAV.

### 3.2. UAV Kinematic Model

In the two-dimensional cooperative path-planning task, all UAVs are homogeneous and share the same kinematic model. A UAV is selected as a representative example. The discrete kinematic equation from time *t* to time t + 1 is given in Equation (1).

(1) $$\left\{\begin{array}{c} x^{t+1}=x^{t}+v_{x}^{t+1}\cdot \Delta t\\ y^{t+1}=y^{t}+v_{y}^{t+1}\cdot \Delta t\\ v_{x}^{t+1}=v_{x}^{t}+a_{x}^{t+1}\cdot \Delta t\\ v_{y}^{t+1}=v_{y}^{t}+a_{y}^{t+1}\cdot \Delta t \end{array}\right.$$

$\left(x^{t},y^{t}\right)$ and $\left(x^{t+1},y^{t+1}\right)$ denote the positions of the UAV at time *t* and time *t* + 1. $\left(v_{x}^{t},v_{y}^{t}\right)\;$ and $\left(v_{x}^{t+1},v_{y}^{t+1}\right)$ denote the velocity components of the UAV along the *x* axis and *y* axis at time *t* and time *t* + 1. $a_{x}^{t+1}$ and $a_{y}^{t+1}$ denote the acceleration components along the *x* axis and *y* axis at time *t* + 1. Δt denotes the simulation time step of the UAV. A smaller value of Δt enables finer control and produces more natural motion. In this study, Δt is set to 0.5 s.

In addition, the flight state constraints of the UAV are given by Equation (2). In Equation (2), $v^{t}=\sqrt{{\left(v_{x}^{t}\right)}^{2}+{\left(v_{y}^{t}\right)}^{2}}$ represents the magnitude of the UAV velocity. $\left(v_{max}\right)$ represents the maximum velocity of the UAV. $a_{max}$ represents the maximum acceleration of the UAV along the *x* axis or *y* axis.

(2) $$\left\{\begin{array}{c} \left|v^{t}\right|\leq v_{max}\\ \left|a_{x}^{t+1}\right|\leq a_{max}\\ \left|a_{y}^{t+1}\right|\leq a_{max} \end{array}\right.$$

## 4. Methodology

This section introduces the proposed multi-UAV cooperative path-planning method. First, a POMDP model for the path planning task is constructed. Then, the network architecture and the improved integrated experience replay mechanism are designed. Finally, a multi-UAV cooperative path-planning algorithm based on IER-MADDPG is proposed.

### 4.1. Design of POMDP Model for Multi-UAV Cooperative Path Planning

The multi-UAV cooperative path-planning task is modeled as a POMDP, which is an extension of the traditional MDP and is used to describe decision-making problems in environments with incomplete information. This model introduces the concepts of observation space and observation function on the basis of the MDP. It can be defined as an eight tuple, $\left\langle N,S,A,P,R,\Omega ,O,\gamma \right\rangle$.

In the POMDP model, *N* represents the number of agents. *S* represents the joint state set of all agents. *A* represents the joint action space. *P* represents the state transition function. $P(s^{\prime }|s,a)$ represents the probability that the state transitions to $s^{\prime }$ after the UAV executes action a in state s. *R* represents the reward function. $\Omega =\left\{\Omega_{1},\Omega_{2},\cdots \;,\Omega_{i},\cdots \;,\Omega_{N}\right\}$ represents the set of agent observation spaces, where $\Omega_{i}$ represents the observation information of UAV i. *O* represents the observation function. $\gamma \in \left[0,1\right]$ represents the discount factor.

(1) Observation Space Design

It is assumed that each UAV is equipped with a GPS and other onboard devices, which can provide its own state information, including position and velocity. The self-state information of UAVs (i) is represented by $\Omega i^{self}\left(t\right)=\left[x_{i}^{t},y_{i}^{t},v{x_{i}}^{t},v_{y_{i}}^{t}\right]$. Communication can be established among UAVs. Therefore, *UAV_i_* can obtain the state information of its neighboring UAVs at time *t*, which is denoted by $\Omega_{i}^{nei}\left(t\right)$. In addition, UAV_i_ needs to obtain information related to the target, such as the relative position and angle, which is denoted by $\Omega_{i}^{tar}$. It also obtains perception information of other entities within its sensing range, including obstacles and neighboring UAVs, which is denoted by $\Omega_{i}^{obs}\left(t\right)$. This information is a necessary condition for effective collision avoidance. Since all UAVs are homogeneous, *UAV_i_* is selected as an example. The observation information of UAV_i_ at time *t* is defined by Equation (3).

(3) $$\Omega_{i}\left(t\right)=\left[\Omega_{i}^{self}\left(t\right),\Omega_{i}^{nei}\left(t\right),\Omega_{i}^{tar}\left(t\right),\Omega_{i}^{obs}\left(t\right)\right]$$

(2) Action Space Design

As described by the kinematic model in Equation (1), the UAV adjusts its velocity by changing the acceleration along the *x* axis and *y* axis in the two-dimensional space, thereby achieving smooth and continuous motion. Therefore, the action space of a single UAV is defined by Equation (4).

(4) $$a_{i}={\left[a_{x_{i}},a_{y_{i}}\right]}^{T}$$

In Equation (4), $a_{x_{i}}$ and $a_{y_{i}}$ represent the acceleration of UAV i along the *x* axis and *y* axis, respectively. To prevent excessive variations in control signals, the upper bound of the absolute value of the acceleration in any direction is set to $a_{max}$.

(3) Reward Function Design

In the multi-UAV cooperative path-planning task, a dense reward mechanism is designed to improve learning efficiency and enable successful completion of the cooperative task. The reward function consists of four components, namely a target guidance reward, collision avoidance reward, step penalty, and task completion reward.

First, the target guidance reward is designed to encourage the UAV to move toward the target. Its formulation is given by Equation (5).

(5) $$r_{tar}^{i}=\left\{\begin{array}{cc} r_{fin}=1, & reached\\ {}[d_{io}\left(t\right)-d_{io}\left(t+1\right)], & otherwise \end{array}\right.$$

In Equation (5), $r_{tar}^{i}$ represents the target guidance reward obtained by UAV i at time step t. $r_{fin}$ represents the arrival reward that UAV i receives when it reaches the designated target position. $d_{io}\left(t\right)-d_{io}\left(t+1\right)$ represents the reduction in the distance between UAV i and the target position after the execution of an action at time t. This reward design alleviates the reward sparsity problem before the UAV reaches the target position and encourages UAV i to approach the target.

Second, task execution in complex environments requires consideration of cooperative safety. Specifically, collisions between UAV i and obstacles, collisions between UAVs, and departures from the task area, which are treated as boundary obstacles, should be avoided. Therefore, the collision avoidance reward is designed as shown in Equation (6).

(6) $$r_{col}^{i}=\left\{\begin{array}{cc} -2, & collision\\ \frac{d_{min}-D}{D}, & otherwise \end{array}\right.$$

In Equation (6), $d_{min}$ represents the minimum distance between UAV_i_ and the surrounding hazardous objects. D represents the maximum detection range of UAV_i_. In addition, a negative step penalty $r_{step}^{i}$ is assigned whenever the UAV executes one action, as shown in Equation (7). The purpose of this reward is to encourage the UAV to explore shorter paths and prevent meaningless movements or stagnation within a local area.

(7) $$r_{step}^{i}=-0.01\;$$

Finally, when all UAVs reach the target position, the task is considered successful. At this time, each UAV receives an additional task completion reward $r_{suc}^{i}$, which has a relatively large value. Its formulation is given by Equation (8).

(8) $$r_{suc}^{i}=5$$

Based on the above reward components, the dense reward function $R_{i}$ of UAV i and the total reward function R of the UAV swarm are defined by Equations (9) and (10).

(9) $$R_{i}=\omega_{1}r_{tar}^{i}+\omega_{2}r_{col}^{i}+\omega_{3}r_{step}^{i}+\omega_{4}r_{suc}^{i}$$

(10) $$R=\sum_{i=1}^{N_{uav}}R_{i}$$

In Equation (9), $\omega_{1}$, $\omega_{2}$, $\omega_{3}$, and $\omega_{4}$ represent the weighting factors of different reward components. These parameters should be fine-tuned through experiments to obtain the optimal cooperative policy.

### 4.2. Network Model Structure Design

**Figure 2:**
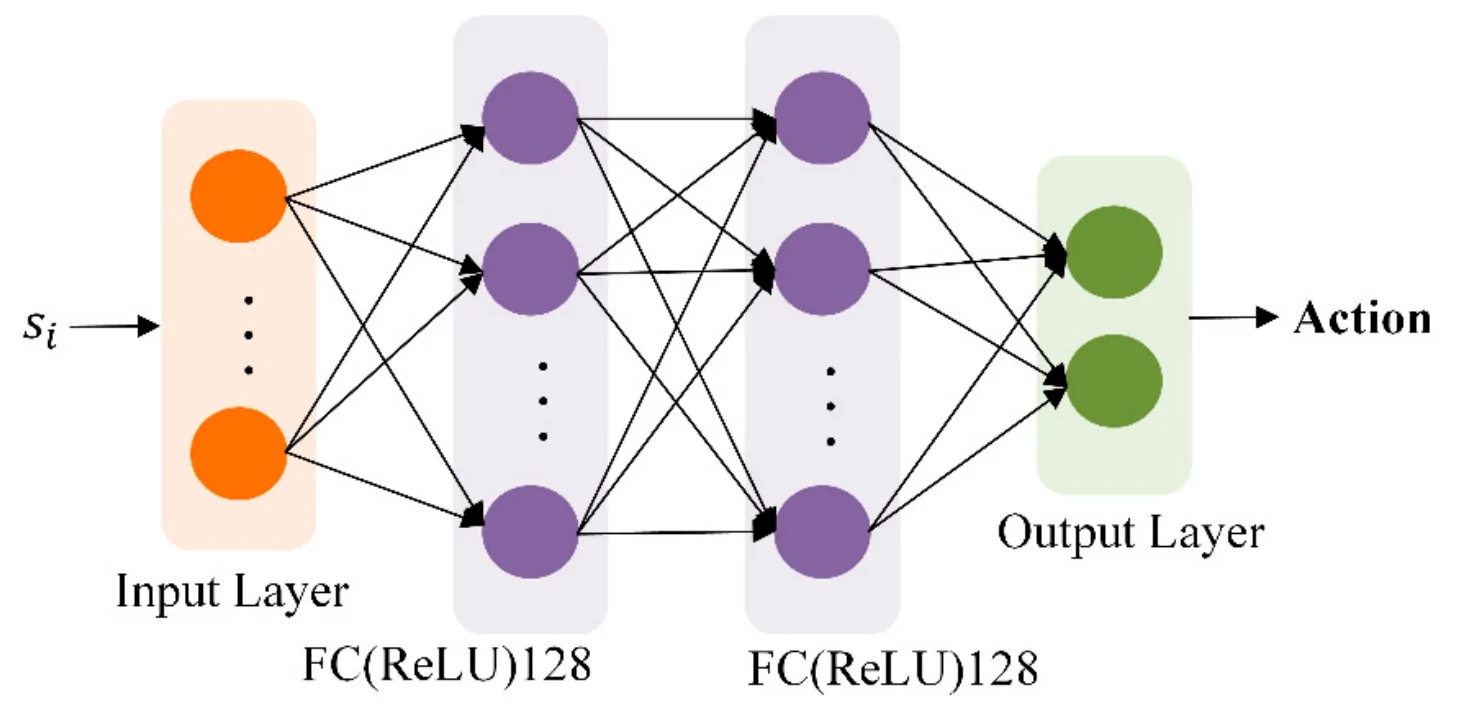
Schematic diagram of action value network.

**Figure 3:**
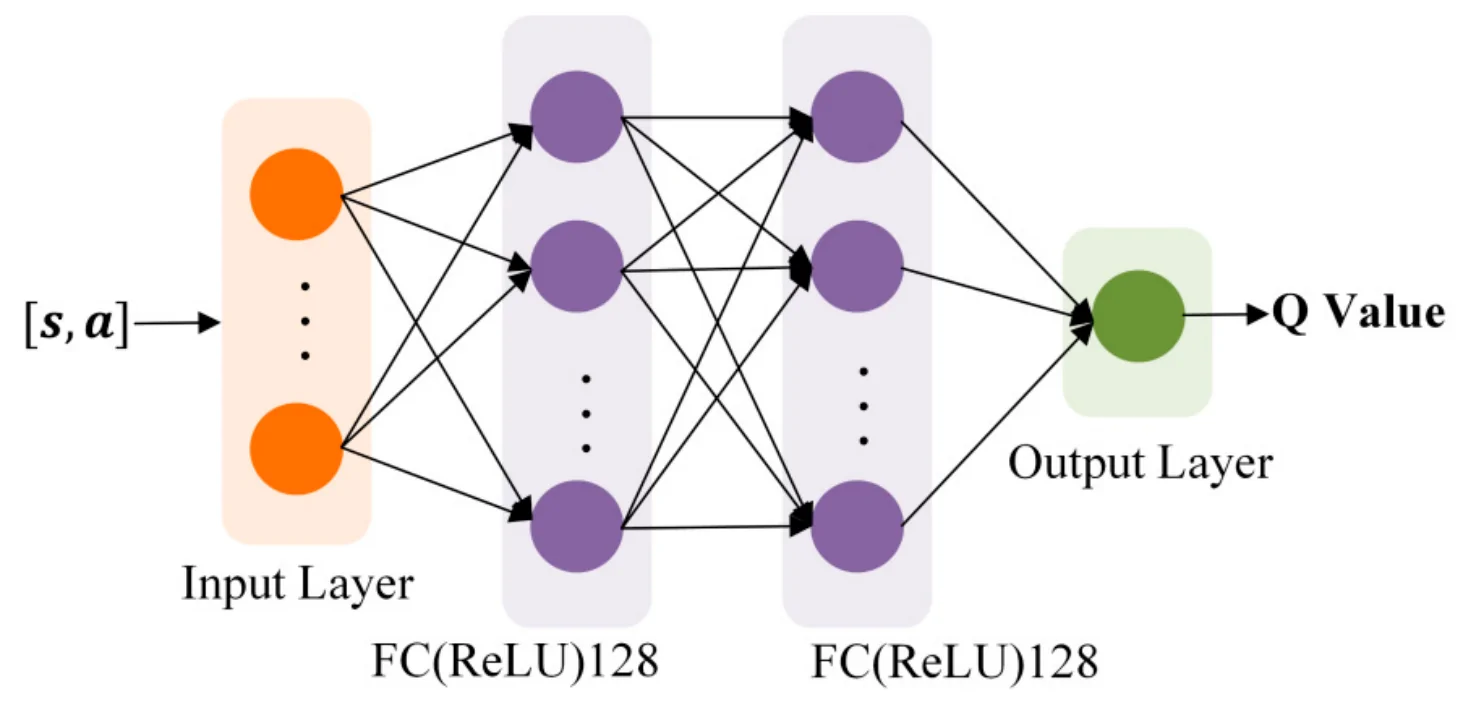
Schematic diagram of critic value network.

The improved MADDPG algorithm is based on the Actor–Critic framework. Each UAV contains an independent Actor network and Critic network, both of which consist of fully connected layers and activation layers. The specific structures are shown in Figure 2 and Figure 3. The input of each Actor network is the local observation information of UAV i, $\Omega_{i}\left(t\right)$. The output is the acceleration of UAV i along the *x* axis and *y* axis. The input of the Critic network is the joint observation of all UAVs, $\Omega =\left\{\Omega_{1},\Omega_{2},\cdots \;,\Omega_{n}\right\}$, and the joint action, $a=\left\{a_{1},a_{2},\cdots \;,a_{n}\right\}$. The output is the Q value of the current state action pair. Both the Actor network and Critic network contain two hidden layers, and each hidden layer contains 128 neurons. A Rectified Linear Unit is adopted as the activation function for all hidden layers. The output layer of the Actor network adopts the Tanh activation function, which maps the acceleration value to the range of $\left[-a_{max},a_{max}\right]$, thereby satisfying the acceleration constraints of the UAV.

### 4.3. Improved Fused Experience Replay Mechanism

**Figure 4:**
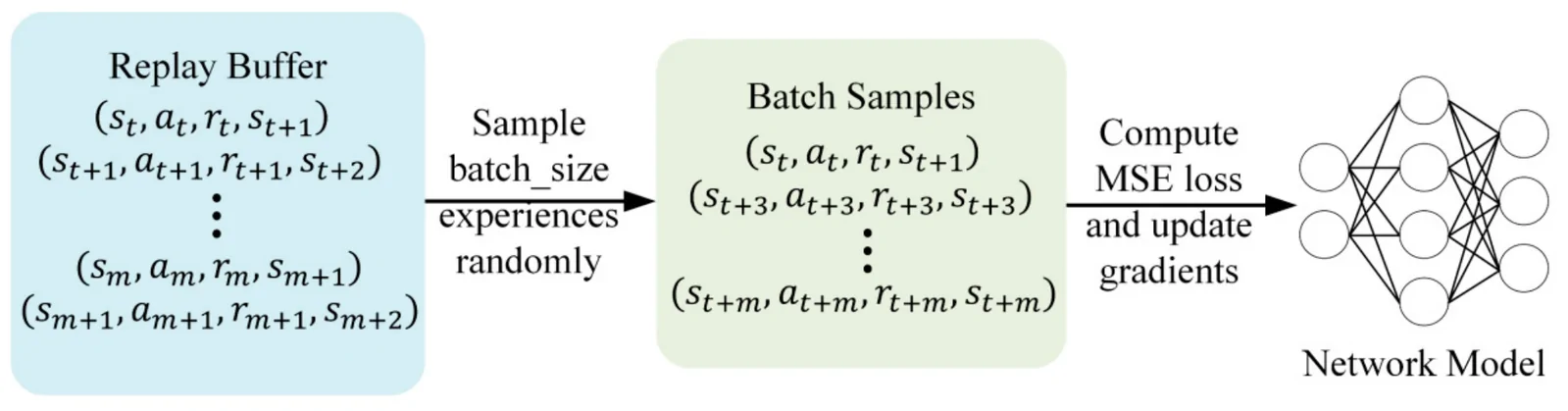
Schematic diagram of random experience replay.

Experience replay is an important technique in reinforcement learning. Its core idea is to store each experience sample, $\left(\left(s_{t},a_{t},r_{t},s_{t+1}\right)\right)$, which is generated through the interaction between the UAV and the environment, into a replay buffer. During training, a batch of samples is selected from the replay buffer to update the network model [31]. This storage mechanism breaks the temporal correlation among samples. As a result, each training batch becomes closer to an independently and identically distributed dataset, which reduces parameter fluctuations and improves training stability. Traditional experience replay adopts random uniform sampling, as shown in Figure 4. This sampling strategy suffers from the problem that high-value samples are diluted by low-value samples. As a result, high-value samples are difficult to sample. Some high-value experiences may even be overwritten or discarded before sufficient learning is achieved, which reduces training efficiency.

**Figure 5:**
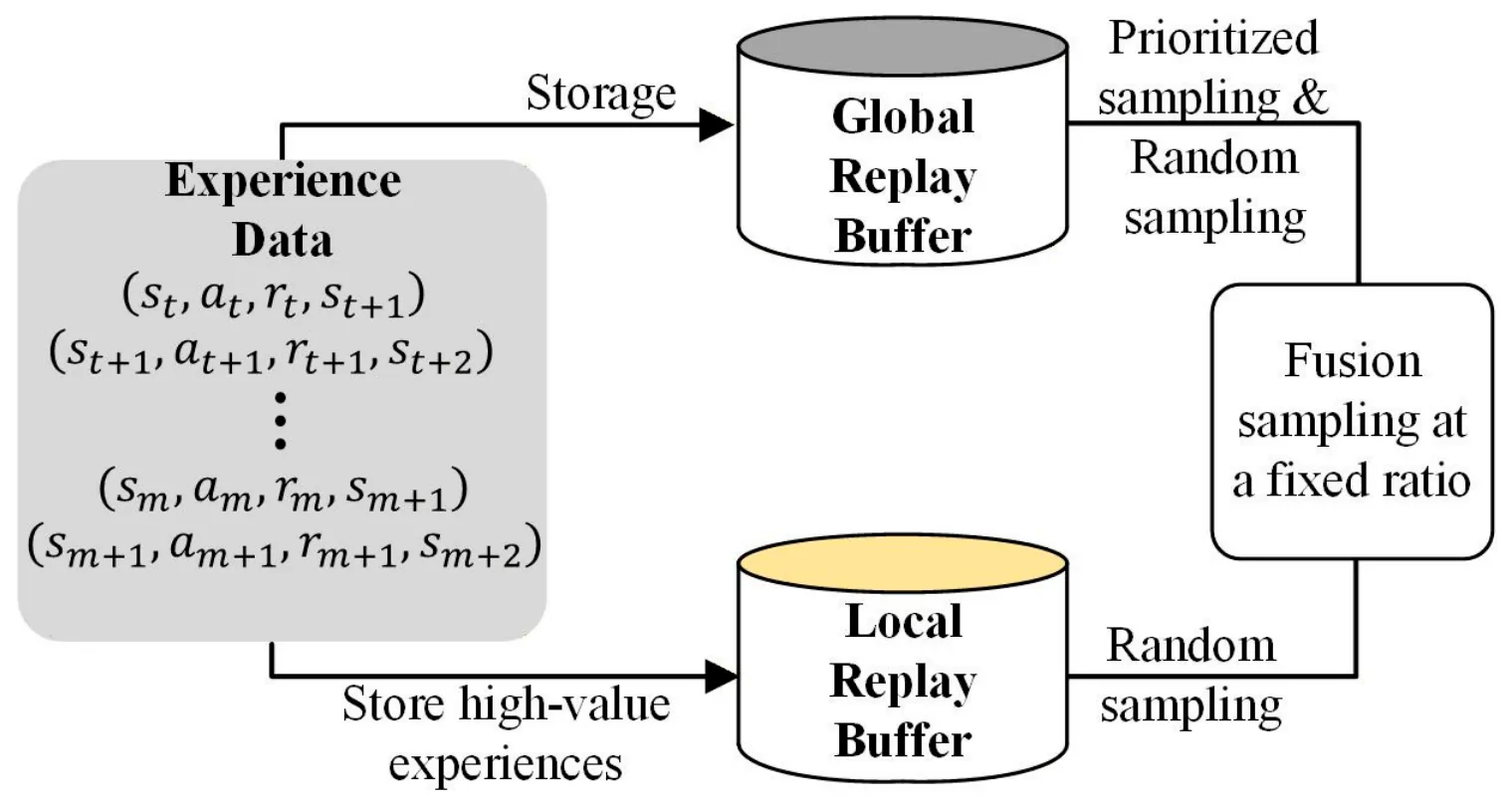
Schematic diagram of fused experience replay.

Therefore, this paper introduces the IER mechanism and designs a dual-layer replay buffer structure, as shown in Figure 5. The first layer is the global replay buffer, which has a large storage capacity and is used to store global experience samples. During sampling from the global replay buffer, a hybrid strategy that combines prioritized sampling and random sampling is adopted. Prioritized sampling evaluates the importance of samples through the Temporal Difference error (TD-error). The TD-error measures the deviation between the predicted Q value and the target value. A larger TD-error indicates greater learning potential of the sample and a higher sampling priority.

For an experience sample $h=\left(s,a,r,s^{\prime }\right)$, the TD-error is calculated by Equation (11).

(11) $$\delta_{h}=r+\underset{a^{\prime }}{max}Q\left(s^{\prime },a^{\prime }\right)-Q\left(s,a\right)$$

The sampling probability of experience sample *h* in the global replay buffer is denoted as $P\left(h\right)$, which is calculated by Equation (12).

(12) $$P(h)=\frac{p_{h}^{\alpha }}{\sum_{k}p_{k}^{\alpha }}$$

(13) $$p_{h}=\left|\delta_{h}\right|+\varepsilon$$

In Equation (12), $p_{h}$ denotes the priority of the experience sample *h*, which is calculated according to the TD-error. *ε* denotes a positive constant with a small value, which prevents the sampling probability of certain samples from becoming zero. α denotes the priority coefficient. When α=0, all experience samples have the same sampling probability, which corresponds to random uniform sampling.

The second layer is the local replay buffer, which has a smaller storage capacity and stores valuable experience samples that are close to the current episode. These samples are obtained from recent episodes that successfully complete the path-planning task. Such experience samples contain key policy information that contributes to task completion and therefore have high learning value. The proposed dual-layer replay buffer mechanism is consistent with the efficient human learning pattern of learning new knowledge while reviewing previous knowledge. The local replay buffer enables the model to learn recent high-value successful experience samples, whereas the global replay buffer preserves and reinforces previously acquired experience. This mechanism promotes rapid learning of new policies and strengthens effective existing experience. As a result, the stability and efficiency of model training are improved.

Specifically, each experience sample generated at a training step is stored in the global replay buffer, while all experiences generated during the current episode are temporarily retained. An episode is considered successful when all UAVs safely reach the target region within the specified number of steps without collision or boundary violation. Experiences from a successful episode are regarded as having relatively high learning value and are stored in the local replay buffer, whereas experiences from an unsuccessful episode are retained only in the global buffer. Therefore, valuable experiences are identified according to whether they originate from a successfully completed episode, without introducing an additional reward or TD-error threshold. During training, the global buffer combines prioritized sampling with random sampling, while the local buffer adopts random sampling. Samples from the two buffers are combined according to a predefined proportion to construct each training batch. When the local buffer reaches its capacity, earlier successful experiences are replaced by more recent successful experiences to maintain sample recency. It should be noted that the fused experience replay buffer \(D\) in Algorithm 1 represents the replay module jointly composed of the global and local buffers rather than a single physical buffer. Algorithm 1 presents the overall training procedure, while the detailed interaction between the two buffers follows the process described in this section.
**Algorithm 1.** Multi-UAV cooperative path planning algorithm based on IER-MADDPGInput: network learning rate *l*, discount factor γ, batch size $N_{\mathrm{batch}}$, training episodes.1: Initialize the policy network $\mu_{i}(s_{i}|\theta_{i}^{\mu })$ and value network $Q_{i}(s_{i},a_{i}|\theta_{i}^{Q})$ for all ${UAV}_{i\in \left\{1,2,\cdots ,n\right\}}$.2: Initialize the target policy network $\mu_{i}^{*}(s_{i}^{\prime }|\theta_{i}^{\mu^{*}})$ for all UAVs, and set $\theta_{i}^{\mu^{*}}\leftarrow \theta_{i}^{\mu }$.3: Initialize the target value network $Q_{i}^{*}(s_{i}^{\prime },a_{i}^{\prime }|\theta_{i}^{Q^{*}})$ for all UAVs, and set $\theta_{i}^{Q^{*}}\leftarrow \theta_{i}^{Q}$.4: Initialize the fused experience replay buffer D.5: for episode=1→E do:
6:        Reset the environment and randomly initialize the initial states of each UAV and the environment.7:        for step=1→T do:
8:              Each UAV generates the corresponding action $a_{i}=\mu_{i}\left(o_{i}\right)$ based on its current local observation $o_{i}$.9:              Add random noise to the action output by the policy network: $a_{i}=\mu_{i}\left(o_{i}\right)+N_{t}$.10:            Execute the joint action $a_{t}=\left\{a_{1},a_{2},\ldots ,a_{n}\right\}$, transit to the next state $s\prime =\left\{s_{1}\prime ,s_{2}\prime ,\ldots ,s_{n}\prime \right\}$, and obtain the current reward set $r_{t}=\left\{r_{1},r_{2},\ldots ,r_{n}\right\}$ and terminal state flag ***d***.11:            Store the experience tuple $\left(s_{t},a_{t},r_{t},s_{t+1},d\right)$ into the experience replay buffer *D*.
12:            ***if***  the number of samples in buffer D reaches the preset threshold:13:                 Sample a batch of data via the fused sampling mechanism and start training.14:            Calculate the value loss function of each UAV according to Equation (15), and update the parameter $\theta_{i}^{Q}$ of the value network.15:            Calculate the policy gradient of each UAV according to Equation (17), and up-date the parameter $\theta_{i}^{\mu }$ of the policy network.

16:            Update the target policy network and target value network of each UAV.                  $\theta_{i}^{\mu^{*}}=\tau \theta_{i}^{\mu }+\left(1-\tau \right)\theta_{i}^{\mu^{*}},\;\;\theta_{i}^{Q^{*}}=\tau \theta_{i}^{Q}+\left(1-\tau \right)\theta_{i}^{Q^{*}};$

17:        *end for*; 18: *end for*;

The dual-layer experience replay mechanism modifies only the experience storage and sampling processes during training and does not change the structures or parameter sizes of the Actor and Critic networks. In this study, both networks retain two hidden layers, with 128 neurons in each layer. The additional training costs mainly arise from maintaining the local replay buffer, calculating TD-errors, and combining prioritized sampling with random sampling. Since the local replay buffer has a smaller capacity than the global replay buffer, its additional storage requirement is limited by its configured capacity. These operations introduce a certain amount of additional computation and storage during training but do not change the main forward- and backward-propagation procedures of the neural networks. During testing and practical inference, the replay buffers and fused sampling mechanism are not involved, and actions are generated directly by the Actor networks from local observations. Therefore, no additional experience-storage or sampling overhead is introduced during inference.

### 4.4. Multi-UAV Cooperative Path-Planning Method Based on IER-MADDPG

**Figure 6:**
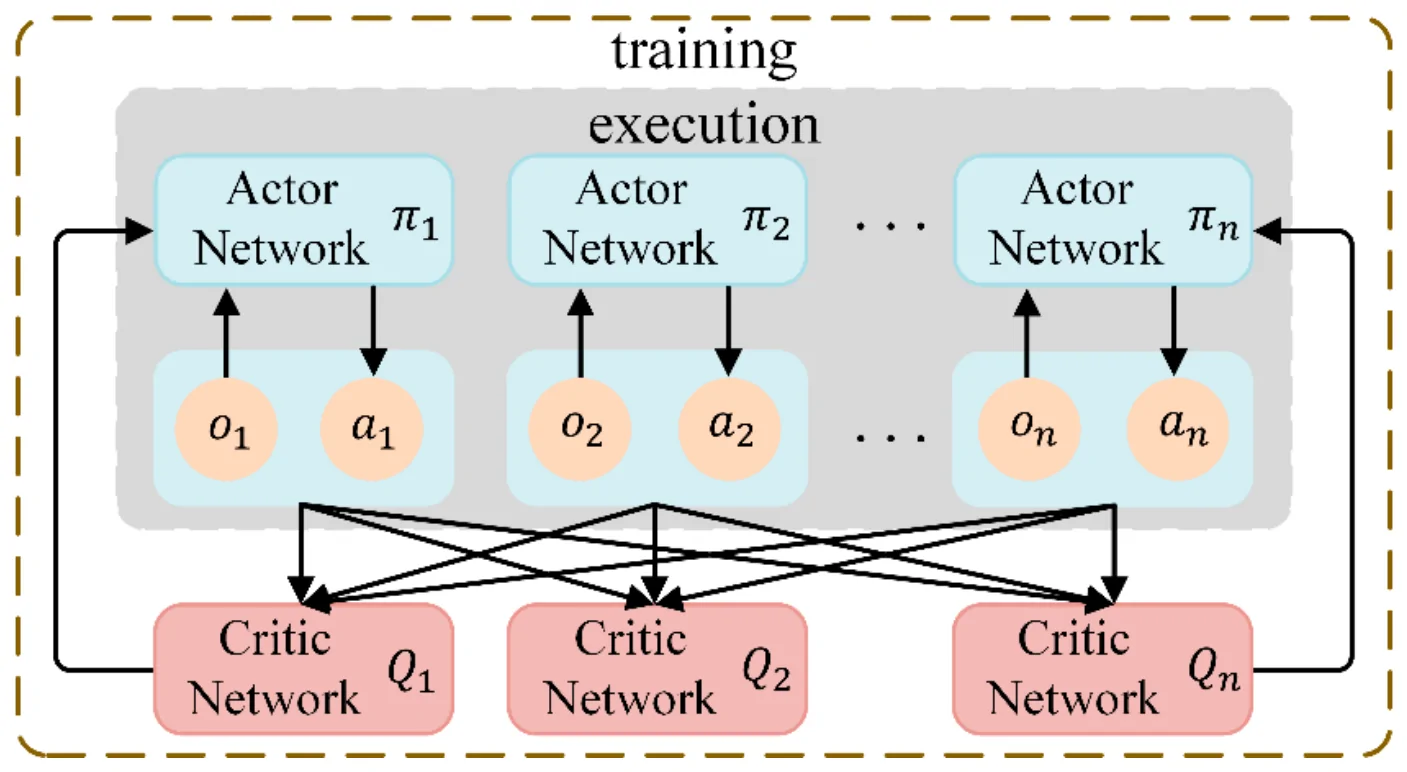
Framework diagram of centralized training and decentralized execution.

Swarm tasks usually involve cooperative interactions between multiple UAVs and the environment. When traditional single-agent reinforcement learning algorithms are applied to such scenarios, problems such as low learning efficiency and unstable training often arise. In a Multi-Agent System, the behavior of each agent affects the states and decisions of other agents, which significantly increases the complexity of the environment [32]. With the development of MARL, numerous MARL frameworks have been studied and applied to various swarm task scenarios. Among them, Centralized Training Distributed Execution is the framework that is the most widely adopted and achieves remarkable performance. Its overall architecture is shown in Figure 6.

Based on the framework, this chapter integrates the experience replay mechanism and the experience sharing mechanism and proposes an IER-MADDPG-based multi-UAV cooperative path-planning method. In this algorithm, each UAV is equipped with an independent Actor network and Critic network. At a certain time during training, UAV_i_ inputs its local observation information $s_{i}$ into its Actor network, which generates the corresponding decision action $a_{i}$. After all UAVs are traversed, the joint action at time t is obtained as $a_{t}=\left\{a_{1},a_{2},\cdots ,a_{n}\right\}$. After the execution of the joint action $a_{t}$, the environment returns the global state information $S^{\prime }$ at the next time step and the reward set of all UAVs, $R=\left\{r_{1},r_{2},\cdots ,r_{n}\right\}$. The Critic network of each UAV is independent and is used to evaluate the action value under its current state. The training process adopts a centralized training strategy. UAVs can communicate with each other to obtain the observation information of other UAVs. The inputs of each Critic network consist of joint observations and joint actions.

**Figure 7:**
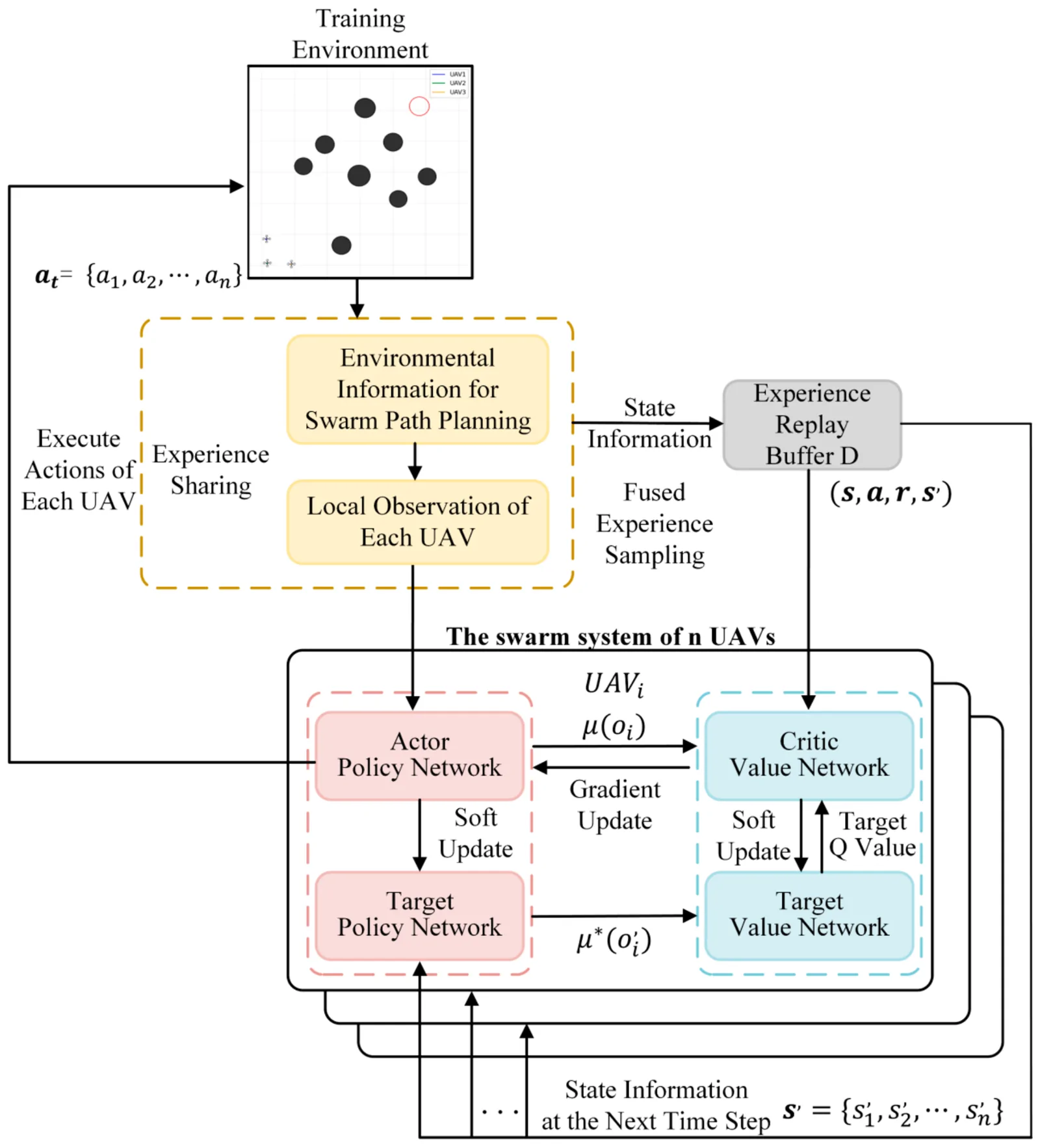
Framework of multi-UAV cooperative path planning method based on IER-MADDPG.

**Figure 8:**
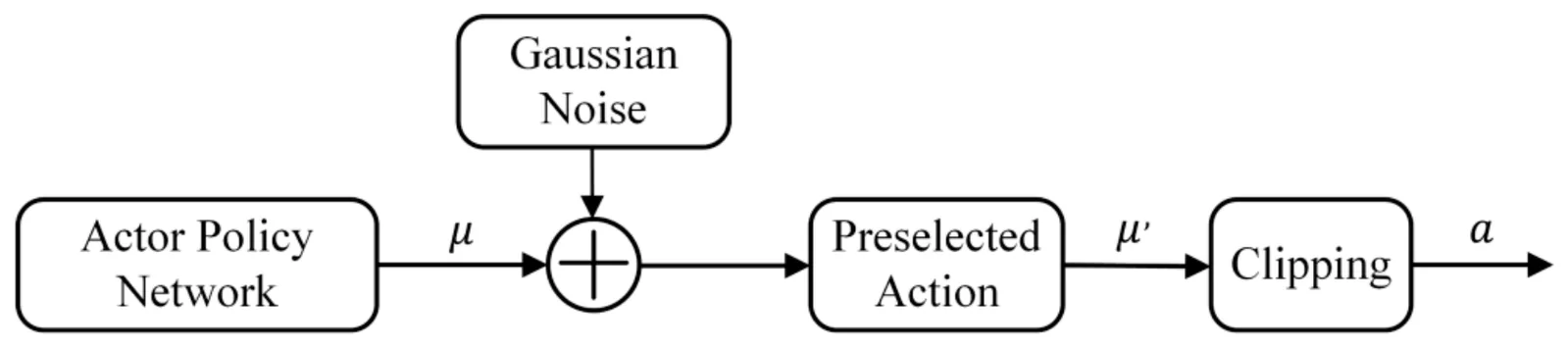
Flowchart of Gaussian noise introduction.

Combined with the overall architecture of the cooperative algorithm, which is shown in Figure 7, the action generation process of a UAV is described as follows. Each UAV processes its local observation information and inputs it into its Actor network. The Actor network generates an optimal raw action under the current experience, but this action is not executed directly. To enhance environmental exploration, action noise is added to the action generated by the Actor network. Finally, the action is clipped according to the range of the action space. As shown in Figure 8, the execution action of UAV_i_ is obtained as $a_{i}$, which is defined by Equation (14).

(14) $$a_{i}=\mu_{i}\left(o_{i}\right)+N_{noise}$$

In Equation (14), $\mu_{i}\left(o_{i}\right)$ denotes the output action of the Actor network of UAV_i_. $N_{noise}$ denotes the random noise that is added to the action and is used to control the environmental exploration behavior of the UAV during different training stages. The Critic network obtains a batch of experience samples through the fusion experience sampling mechanism and calculates the state action value $Q\left(s,a\right)$ of each sample.

During training, a relatively stronger Gaussian noise is used at the early stage to encourage broad exploration of the environment and action space. As training progresses, the noise intensity is gradually reduced so that the policy shifts from exploration to stable exploitation. The noise intensity is selected according to the scale of the bounded action space, and the perturbed action is clipped to satisfy the UAV acceleration constraints. Gaussian noise is used only during training and is disabled during testing and practical inference.

In addition, a target Actor network and a target Critic network are introduced to reduce the variance of value estimation and avoid oscillations during training. The target networks share the same architecture as the corresponding online networks. The target Actor network generates the next action $a^{\prime }$ according to the next state $s^{\prime }$ in the experience sample. The state $s^{\prime }$ and action $a^{\prime }$ are then input into the target Critic network to obtain the value estimation of the next state action pair, $Q^{*}\left(s^{\prime },a^{\prime }\right)$. The parameters ${\theta_{i}}^{Q}$ of the Critic network are updated by minimizing the error between the predicted *Q* value of the Critic network and the target *Q* value. The loss function is defined by Equations (15) and (16).

(15) $$L\left(\theta_{i}^{Q}\right)=\frac{1}{N}\sum_{j}{\left[Q\left(s,a|\theta_{i}^{Q}\right)-y\right]}^{2}$$

(16) $$y=r+\gamma Q^{*}\left(s^{\prime },a^{\prime }|\theta_{i}^{Q^{*}}\right)$$

In Equations (15) and (16), *N* denotes the batch size that is sampled from the fusion replay buffer during each update. $s=\left\{s_{1},s_{2},\cdots ,s_{n}\right\}$ denotes the state information of all UAVs at the current time step. $a=\left\{a_{1},a_{2},\cdots ,a_{n}\right\}$ and $a^{\prime }=\left\{a_{1}^{\prime },a_{2}^{\prime },\cdots ,a_{n}^{\prime }\right\}$ denote the joint actions of all UAVs at the current time step and the next time step. The Actor network of UAV_i_ optimizes its network parameters ${\theta_{i}}^{u}$ by maximizing the value obtained from the current action. The policy gradient is defined by Equation (17).

(17) $$\nabla_{\theta_{i}^{\mu }}J\approx \frac{1}{N}\sum_{j}\nabla_{a_{i}}Q(s,a|{\theta_{i}}^{Q})\nabla_{\theta_{i}^{\mu }}\mu \left(o_{i}\right)$$

To ensure stable convergence during training, the target Actor network and target Critic network of UAV_i_ adopt a soft update mechanism. During each update, the parameters of the target networks are adjusted by a small amount toward the parameters of the corresponding online networks. The update process is defined by Equations (18) and (19).

(18) $$\theta_{i}^{Q^{*}}=\tau \theta_{i}^{Q}+\left(1-\tau \right)\theta_{i}^{Q^{*}}$$

(19) $$\theta_{i}^{\mu^{*}}=\tau \theta_{i}^{\mu }+\left(1-\tau \right)\theta_{i}^{\mu^{*}}$$

In Equations (18) and (19), $\theta_{i}^{Q}$ and $\theta_{i}^{\mu }$ denote the parameters of the Critic network and Actor network of UAV_i_, respectively. $\theta_{i}^{Q^{*}}$ and $\theta_{i}^{\mu^{*}}$ denote the parameters of the target Critic network and target Actor network of UAV_i_. τ denotes the soft update coefficient, which is usually assigned a small value and is commonly set to 0.01. The overall algorithm procedure is presented in Algorithm 1.

## 5. Experimental Verification and Result Analysis

### 5.1. Experimental Scenarios and Parameter Settings

In this section, the experimental environment is established on the Windows 11 operating system equipped with NVIDIA GeForce RTX 2080Ti, and Python 3.8.19 was used to build a simulation platform for multi-UAV cooperative path planning. The training scenario is a two-dimensional airspace environment with a fixed altitude, as shown in Figure 8.

**Figure 9:**
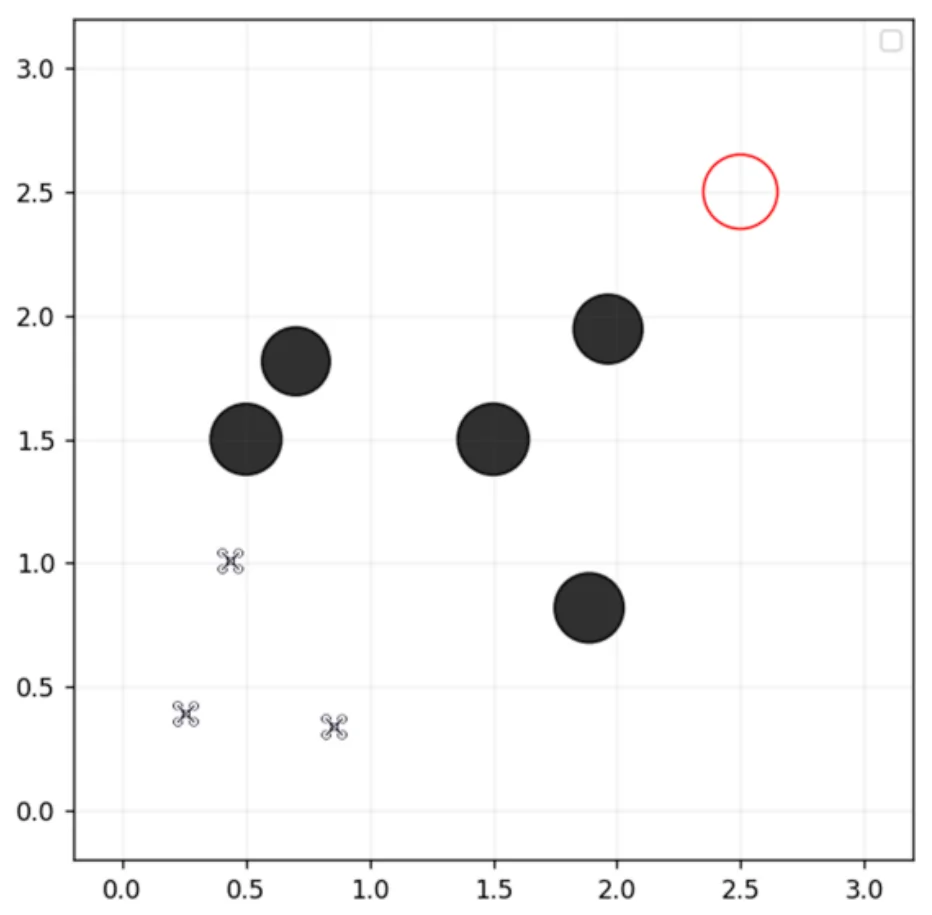
Simulation environment for multi-UAV swarm control.

In Figure 9, the red circle represents the target position of the UAV swarm, which serves as the assembly area. The black solid circles represent hazardous areas, such as obstacles and no fly zones. Before each training episode, the initial positions of the UAV swarm, as well as the positions and sizes of all obstacles, are generated randomly within predefined ranges.

In addition, constraints are imposed on the motion parameters of the UAVs to ensure the rationality and realism of the experiments. The maximum flight speed is set to 10 m/s, and the maximum acceleration is set to $4\;m/s^{2}$. Based on the above settings, the IER-MADDPG algorithm proposed in Section 4.4, the MADDPG algorithm, and the IL-DDPG algorithm are employed to conduct multi-UAV cooperative path-planning training. Through multiple comparative training experiments, the hyperparameters of the algorithms and the weight parameters of the reward function are determined. The corresponding settings are listed in Table 1 and Table 2.

After the training process is completed, multidimensional evaluation metrics are required to assess the performance of the model. The primary objective of the multi-UAV cooperative path-planning task in this section is to guide all UAVs to reach the target position safely with the shortest completion time and the optimal path. Therefore, the following evaluation metrics are designed to quantitatively analyze the effectiveness and superiority of the proposed method.

(1) Success rate (SR): This metric evaluates the effectiveness of task completion. In Equation (20), $N_{s}$ denotes the number of successful episodes during training and testing, and $N_{t}$ denotes the total number of episodes. SR is defined by Equation (20).

(20) $$SR=\frac{N_{s}}{N_{t}}$$

(2) Collision rate (CR): This metric evaluates the safety of task execution and represents the probability that a UAV collides with an obstacle or another UAV. In Equation (21), $N_{c}$ denotes the number of collision episodes. CR is defined by Equation (21).

(21) $$CR=\frac{N_{c}}{N_{t}}$$

(3) Average arrival time (AAT): This metric evaluates task completion efficiency. It is defined as the average number of time steps that the UAV swarm requires to complete the path-planning task in successful episodes. A smaller AAT indicates higher task completion efficiency. In Equation (22), $N_{s}$ denotes the number of successful episodes. AAT is defined by Equation (22).

(22) $$AAT=\frac{\sum_{i=0}^{i=N_{s}}t_{i}}{N_{s}}$$

(4) Average flight distance (AFD): This metric evaluates the path optimization performance. A shorter flight distance corresponds to lower energy consumption. In Equation (23), $D_{k,i}=\int_{0}^{t_{f,k}}\left\|v_{k,i}\right\|dt$ denotes the flight distance of UAV i in a specific episode, and $N_{uav}$ denotes the number of UAVs that participate in the task. AFD is defined by Equation (23).

(23) $$AFD=\frac{\sum_{k=1}^{N_{s}}\sum_{i=1}^{N_{uav}}D_{k,i}}{N_{s}\cdot N_{uav}}$$

(5) Velocity consistency (VC): This metric evaluates the cooperative performance of the UAV swarm. Its value ranges from 0 to 1. A value closer to 1 indicates better velocity consistency among UAVs, which can effectively reduce conflicts and collisions.

(24) $$VC=\frac{\left\|v_{i}+\sum_{j\in N_{i}}v_{j}\right\|}{\left\|v_{i}\right\|+\left\|\sum_{j\in N_{i}}v_{j}\right\|}$$

### 5.2. Training Process Visualization and Analysis

**Figure 10:**
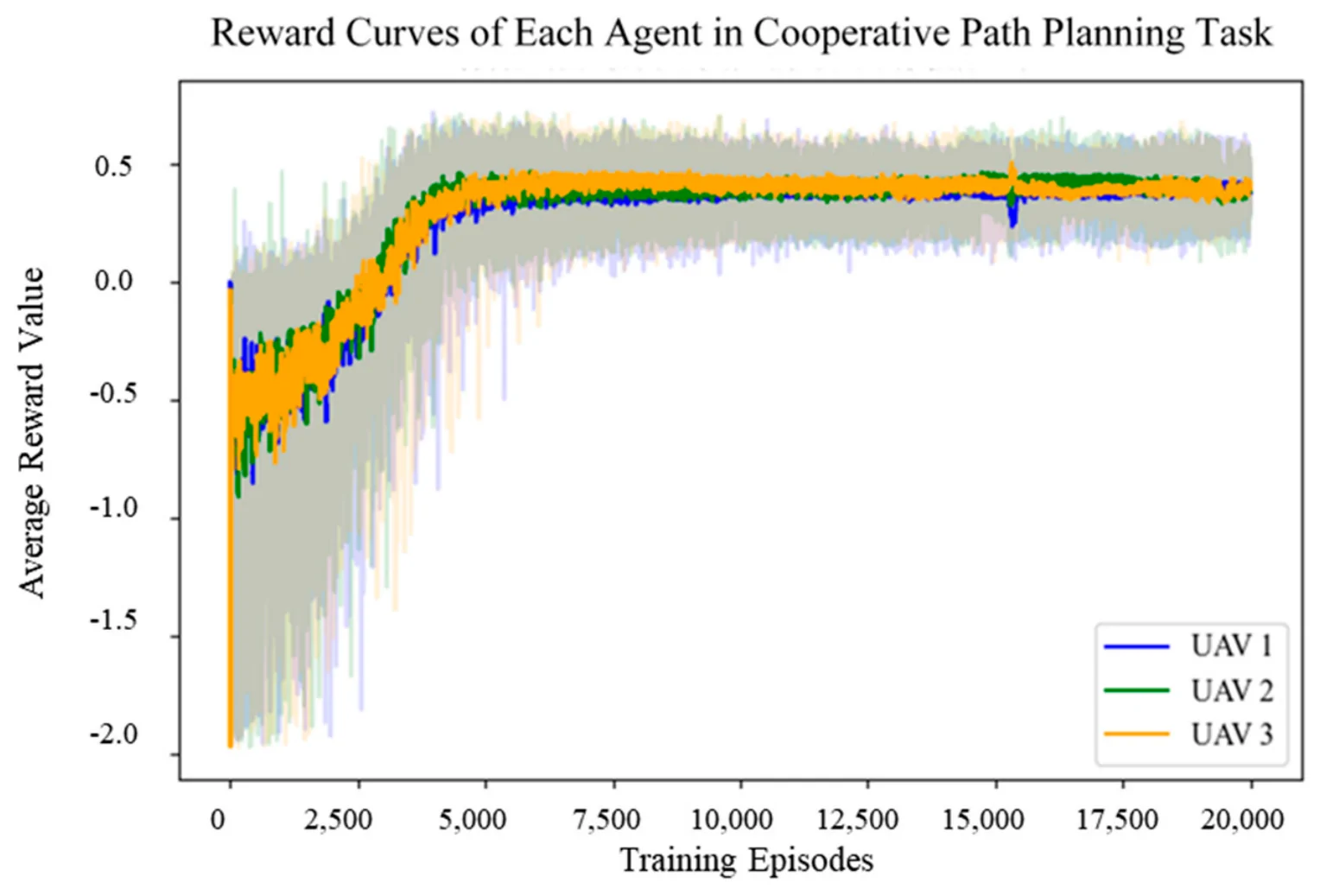
Reward curves of each UAV in training scenarios.

**Figure 11:**
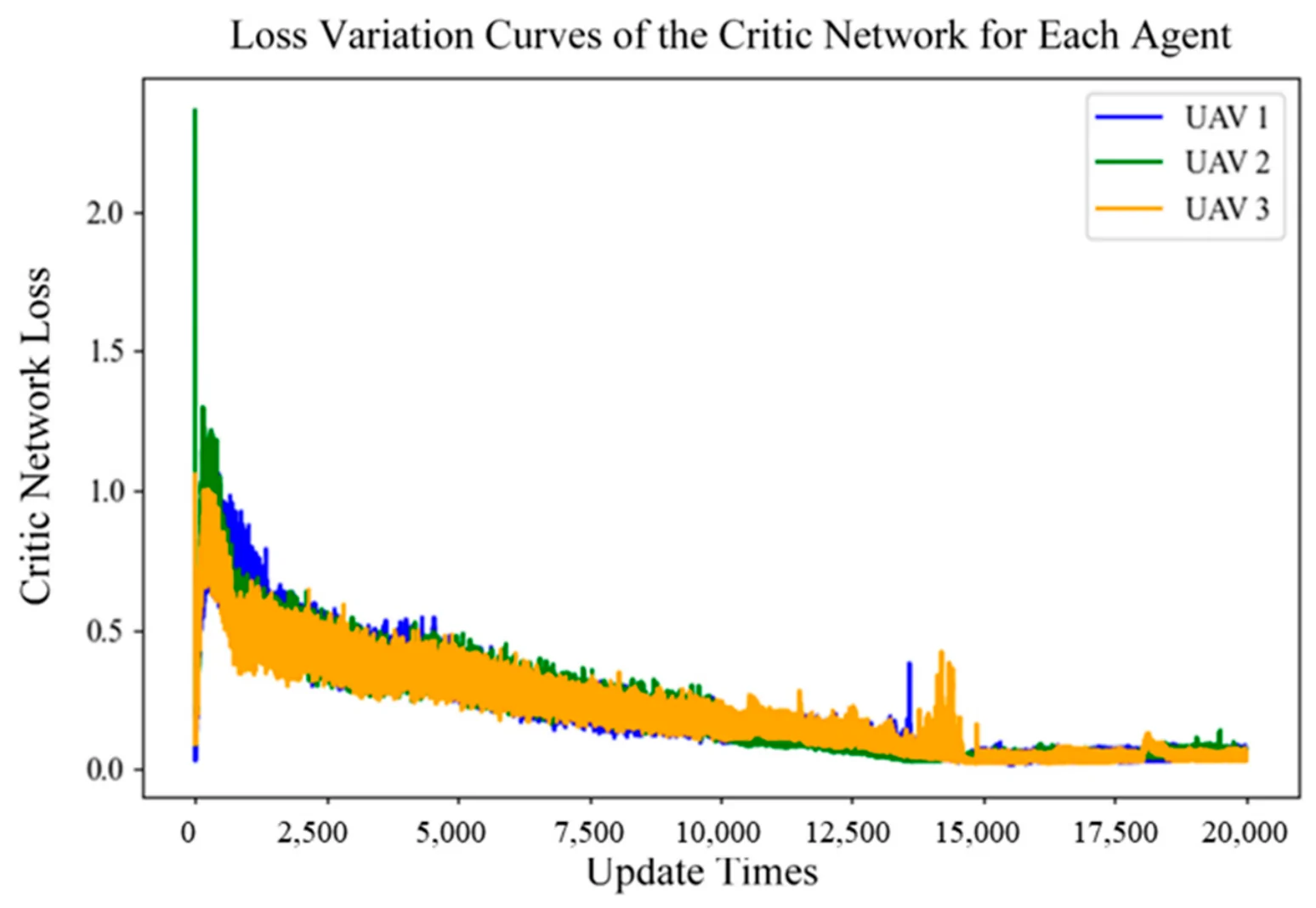
Loss variation curves of value networks for each UAV.

Under the customized simulation environment and predefined parameter settings, this section first constructs a training scenario to verify the convergence characteristics of the proposed method. Subsequently, algorithm effectiveness analysis, comparative experiments, and generalization tests were conducted. The training scenario consists of three UAVs and four randomly distributed obstacles. To ensure the safety of multi-UAV cooperative path planning, a safe distance must be maintained between UAVs and between UAVs and obstacles to avoid collisions. The maximum number of training episodes was set to 20,000, and the complete training process was conducted for 20,000 episodes, as shown in Figure 10 and Figure 11. Figure 10 presents the reward variation over the complete 20,000 training episodes, whereas Figure 11 presents the corresponding Critic-network optimization process, with update times shown on the horizontal axis. 

As shown in Figure 10, during the initial training stage, from 0 to 3500 episodes, the reward values of all UAVs increase rapidly from a low range around −2, accompanied by significant fluctuations. This result indicates that the IER-MADDPG method can actively explore the environment and learn effective cooperative strategies during the early stage. After 3500 episodes, the reward fluctuations become significantly narrower and gradually converge to a stable range between 0 and 0.5. This result indicates that the UAV swarm has learned a near optimal cooperative path-planning strategy and can complete tasks stably in complex obstacle environments. Meanwhile, the final reward values of the three UAVs tend to be consistent, which indicates that a stable benefit allocation has been achieved among UAVs. This result provides preliminary evidence for the cooperative capability of the proposed algorithm. The Critic loss curves further explain the training mechanism of the algorithm. During the initial training stage, the loss remains within a relatively high range, between 1.5 and 2.0, and exhibits substantial fluctuations. This phenomenon occurs because the actions contain strong randomness during the exploration stage, which leads to large estimation errors of the value function for joint state action pairs. As training progresses, the Critic losses of all UAVs decrease rapidly and converge to a stable range close to zero. This result indicates that the value function under the centralized training strategy can provide accurate value evaluation. It can also predict future rewards more accurately and provide reliable guidance for the optimization of the Actor network.

### 5.3. An Effectiveness Analysis of the Algorithm in Multiple-Obstacle Scenarios

To verify the effectiveness of the proposed method, simulation experiments were conducted in scenarios with different numbers of obstacles. The numbers of obstacles were set to 5, 6, 7, and 8, respectively. The initial positions of UAVs, the initial positions of obstacles, and the center of the target area were randomly generated within a specified range. This group of experiments focused on verifying the effectiveness of IER-MADDPG in completing cooperative path-planning tasks under different obstacle configurations. It is different from the comparative experiments presented in Section 5.4.

**Figure 12:**
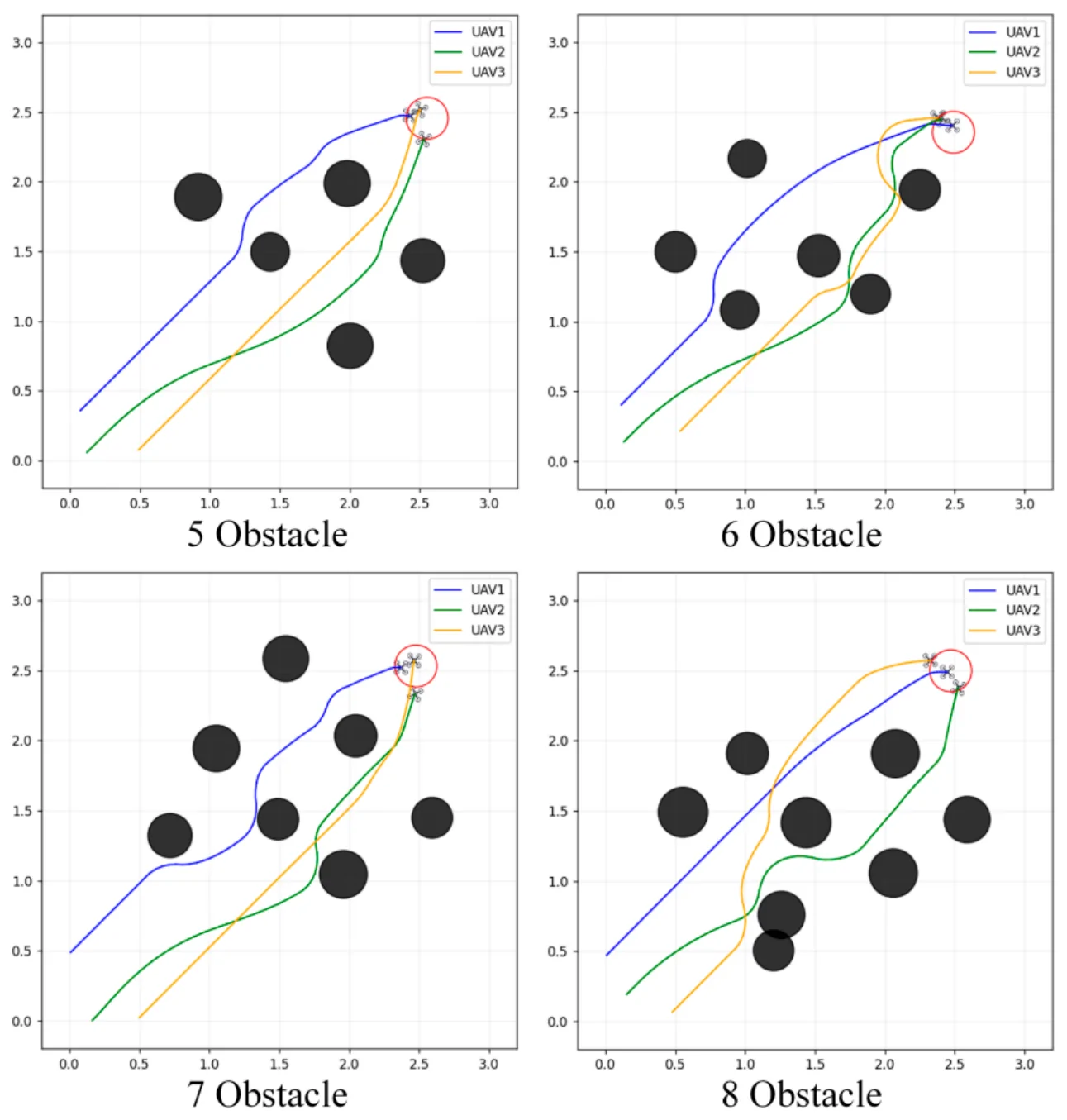
Path of three-UAV cooperative tasks in obstacle environments.

As shown in Figure 12, in task scenarios that contain 5 to 8 obstacles, the three UAVs can depart from their initial positions, avoid all obstacles, and cooperatively reach the target area, which is represented by the red circle. As the number of obstacles increases, the UAV swarm can still generate smooth and collision-free paths. This result indicates that the IER-MADDPG algorithm possesses strong environment perception and obstacle avoidance capabilities. Meanwhile, the three trajectories maintain relatively stable spacing throughout the flight process. This result reflects that the algorithm can achieve cooperation among agents through the centralized value function under the distributed execution framework. It also avoids local optima and path conflicts.

### 5.4. Comparative Experimental Results and Analysis

**Figure 13:**
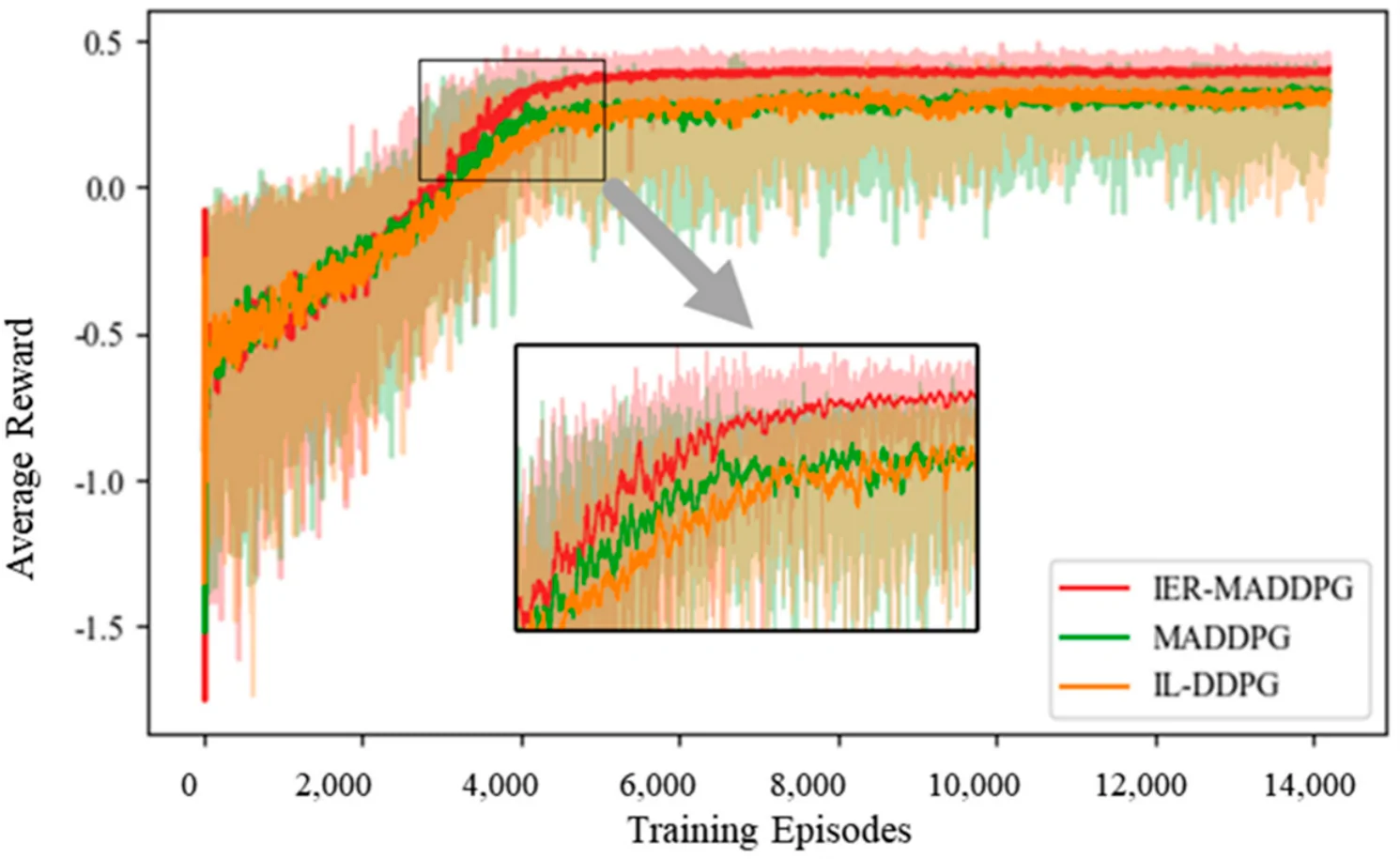
Curve of average reward per episode.

**Figure 14:**
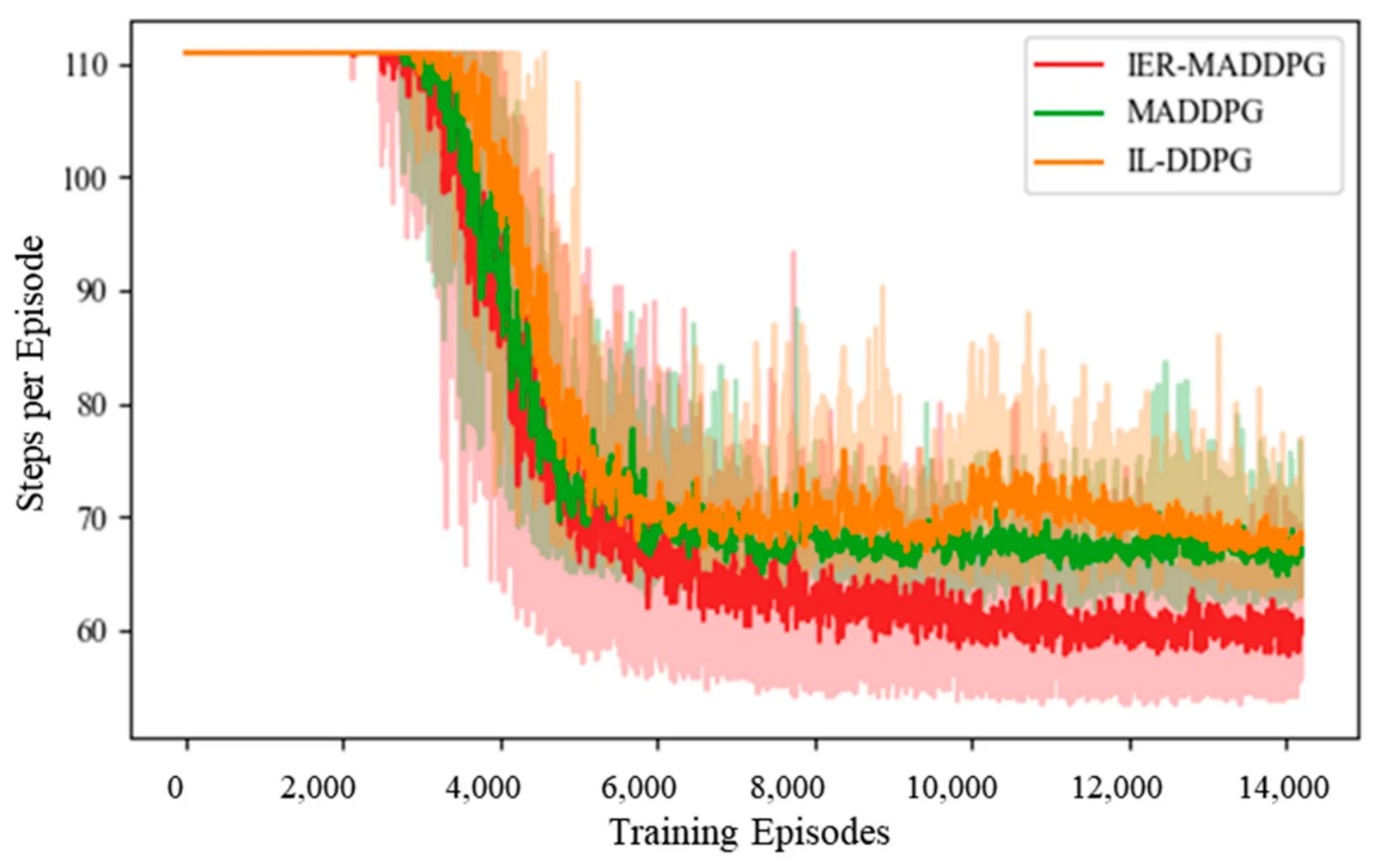
Curve of completed steps per episode.

The comparative curves in Figure 13 and Figure 14 are displayed for up to 14,000 episodes because the algorithms had already exhibited stable convergence trends. Comparative experiments were conducted between the proposed method, MADDPG, and IL-DDPG. As shown in Figure 13, the IER-MADDPG algorithm begins to converge at approximately 4000 episodes, whereas MADDPG and IL-DDPG gradually converge after 4500 episodes. The IER-MADDPG algorithm not only achieves a faster convergence speed but also maintains a higher average reward after convergence than the other two algorithms. In addition, the reward curve of IER-MADDPG exhibits smaller fluctuations during the later training stage.

As shown in Figure 14, the number of steps required to complete each episode under IER-MADDPG decreases rapidly during the initial training stage and stabilizes within a relatively low range, between 60 and 70. This result indicates that the learned policy can guide UAVs to the target area efficiently. The episode completion step curves of MADDPG and IL-DDPG decrease more slowly, and noticeable fluctuations still exist during the later training stage. Meanwhile, the number of steps required by IER-MADDPG remains lower than those of MADDPG and IL-DDPG during the later training stage. This result demonstrates that the proposed method can improve policy search efficiency, suppress training oscillations, and enhance training stability and sample utilization efficiency.

Under the same training environment and parameter settings, each interaction step generates and stores a corresponding experience sample. Therefore, the number of training episodes required to reach a stable reward level and the episode completion steps can serve as quantitative indicators of the utilization efficiency of environmental interaction experiences. As shown in Figure 13, IER-MADDPG begins to converge at approximately 4000 episodes, whereas MADDPG and IL-DDPG gradually converge after 4500 episodes. Moreover, as shown in Figure 14, the episode completion steps of IER-MADDPG decrease rapidly and stabilize between 60 and 70 steps, remaining lower than those of the other two algorithms. These results indicate that IER-MADDPG can learn a stable and effective cooperative path-planning policy with fewer training episodes and lower episode completion steps, thereby providing quantitative comparative evidence of its improved sample utilization efficiency.

To further verify the superiority of the proposed method, MAPPO and MATD3 were added as comparison algorithms. The success rate, collision rate, average arrival time, average flight distance, and velocity consistency of the five algorithms were evaluated over 2000 test episodes under different obstacle scenarios. Table 3 presents the results of the five methods in environments containing 5, 7, and 9 obstacles.

Unlike the effectiveness experiments in Section 5.3, the comparative experiments select environments containing 5, 7, and 9 obstacles to evaluate the performance differences among IER-MADDPG, MADDPG, IL-DDPG, MAPPO and MATD3 under different levels of environmental difficulty.

In the environment with 5 obstacles, IER-MADDPG achieves a success rate of 92.55%, which is comparable to the 92.60% value achieved by MAPPO and higher than those of MADDPG, IL-DDPG, and MATD3. Although the success rate of MAPPO is marginally higher by 0.05 percentage points, the average arrival time and average flight distance of IER-MADDPG are 71.70 and 336.04, respectively, which are considerably lower than the corresponding values of 84.98 and 387.67 obtained by MAPPO. Moreover, IER-MADDPG achieves the highest velocity consistency of 0.971. These results demonstrate that IER-MADDPG maintains a high task success rate while providing better task efficiency and multi-UAV coordination.

As the number of obstacles increases, IER-MADDPG exhibits a more evident overall advantage. In the environment with 9 obstacles, its success rate remains at 85.15%, exceeding the 79.25% value of MAPPO, 75.00% of MADDPG, 50.55% of IL-DDPG, and 41.10% of MATD3. Its collision rate is 14.85%, which is lower than those of all comparison algorithms. In terms of task efficiency and coordination, IER-MADDPG achieves the lowest average arrival time and average flight distance and the highest velocity consistency in all three environments. Overall, IER-MADDPG provides a better balance among task success, safety, path efficiency, and multi-UAV coordination.

**Figure 15:**
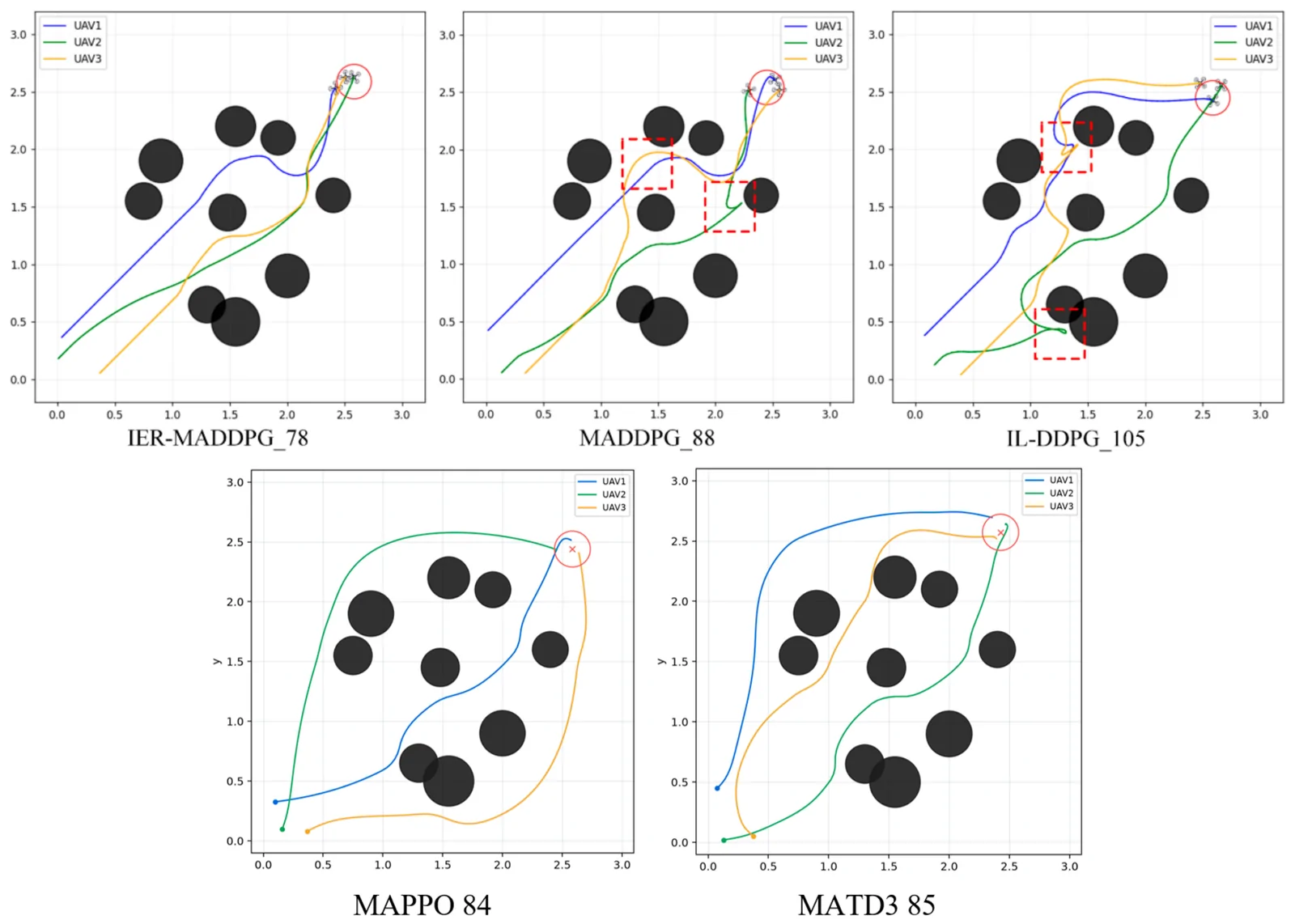
Trajectories of UAV swarm under different cooperative strategies.

In the task scenario containing nine randomly distributed obstacles, the cooperative trajectories generated by IER-MADDPG, MADDPG, IL-DDPG, MAPPO, and MATD3 are shown in Figure 15. The five strategies require 78, 88, 105, 84, and 85 time steps, respectively, to complete the swarm task. Under IER-MADDPG, the UAV swarm travels from the starting area to the target area without obvious detours and performs only minor heading adjustments near the three adjacent obstacles on the right side. The three UAVs eventually reach the target area almost simultaneously and in an orderly manner. The trajectories generated by MADDPG are generally smooth. However, some UAVs, such as Trajectory 2, exhibit slight obstacle-avoidance redundancy near the single obstacle on the left side. Although no collision occurs, the flight distance is increased. IL-DDPG exhibits an evident lack of coordination: two UAVs, corresponding to Trajectories 1 and 3, perform crossing obstacle-avoidance maneuvers in the central obstacle cluster, while Trajectory 2 exhibits repeated obstacle avoidance near the single obstacle in the lower-right region, resulting in considerable increases in AAT and AFD.

MAPPO completes the task within 84 time steps, but its trajectories still contain some redundant detours. UAV2 performs a relatively large arc-shaped detour above the obstacles, while UAV3 first travels along the lower part of the environment and then approaches the target from the right side, resulting in noticeable differences among the UAV path lengths. MATD3 requires 85 time steps to complete the task. UAV1 climbs along the left side of the environment and performs a relatively long detour above the obstacles. UAV3 initially turns toward the lower-left region and subsequently undergoes multiple heading adjustments around the central obstacles, resulting in a more winding trajectory. In comparison, IER-MADDPG generates more compact trajectories, performs only necessary local adjustments near the obstacles, and completes the task using the fewest time steps, further demonstrating its advantages in multi-UAV cooperative obstacle avoidance and path-planning efficiency.

### 5.5. Generalization Experiments and Result Analysis

The generalization capability of the learned multi-UAV cooperative path-planning strategy is further evaluated in this section. The maximum episode length is set to 110 time steps. At the beginning of each episode, the initial positions of the UAV swarm, the target area, and the obstacle locations are randomly generated within a specified region. Two types of generalization experiments were conducted. First, the number of UAVs is expanded to 4, 5, and 6 based on the trained neural network parameters. Second, random static obstacles in the training environment are gradually replaced by dynamic obstacles, and the task completion performance of the UAVs was recorded. According to the performance metrics described in Section 5.2, 2000 test episodes were conducted for each scenario.

As shown in Table 4, in environments with a fixed number of obstacles, the SR decreases from 86.90% to 73.90% as the number of UAVs increases from 4 to 6, while the CR increases accordingly. This result indicates that the complexity of multi-UAV cooperation increases with the scale of the UAV swarm.

**Figure 16:**
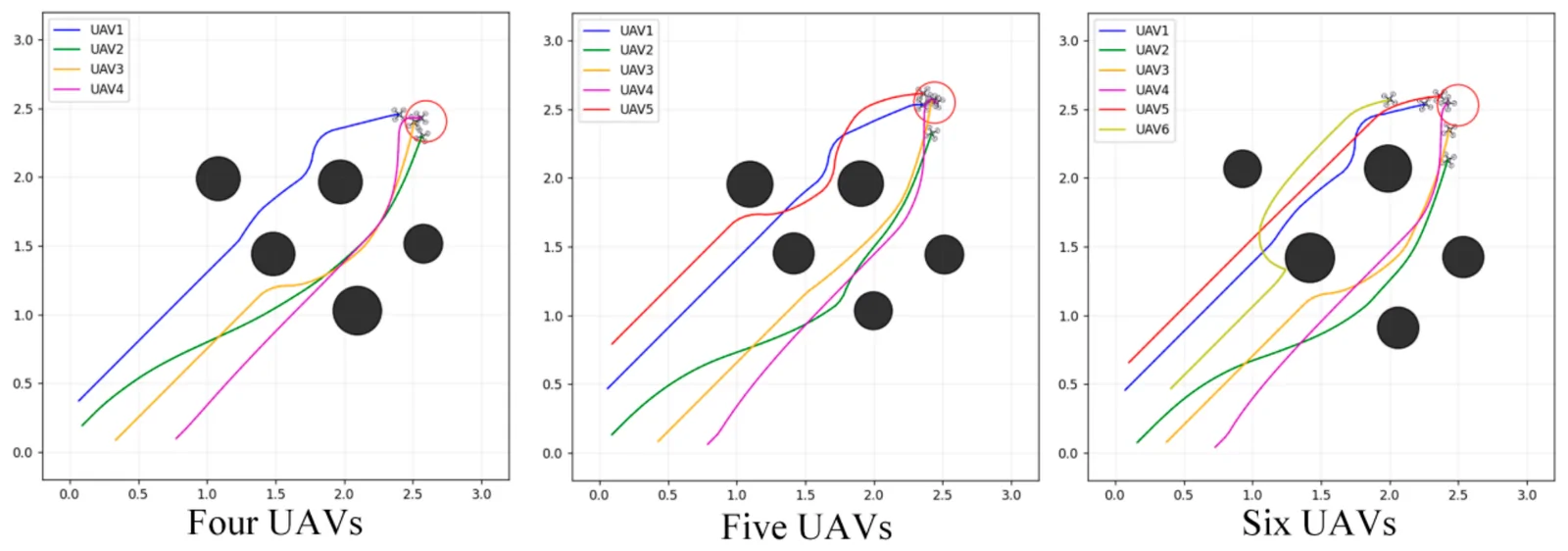
Trajectories under different numbers of UAVs.

However, even in the scenario with six UAVs, the SR remains above 70%. This result demonstrates that the MADDPG-based centralized training framework possesses a certain degree of scalability. The cooperative strategy that is achieved through global information sharing can still maintain its effectiveness when the swarm size increases. The AAT remains approximately 71.8 time steps under different scales and exhibits little variation. This result indicates that the increase in swarm size does not significantly affect overall task execution efficiency. The VC remains above 0.975 in all scenarios, which reflects that the algorithm can still maintain good formation coordination and velocity coordination during multi-UAV cooperative motion control. The test results are shown in Figure 16. This result further demonstrates the advantage of the centralized optimization strategy adopted by IER-MADDPG.

In the final generalization experiment, the number of UAVs is set to 3, and the number of random obstacles is set to 5. Static obstacles are gradually replaced with dynamic obstacles, which means that the number of dynamic obstacles increases progressively. At the beginning of each test episode, each dynamic obstacle was assigned a randomly generated motion direction and moved at a constant velocity along that direction. The velocity is set to 0.8 m/s. The test results are presented in Table 5.

As shown in Table 5, the SR of the UAV swarm decreases from 85.6% to 66.25% as the number of dynamic obstacles increases. Compared with the task SR under the environment with completely static obstacles, which is 92.55% according to Table 2, the SR under the environment with completely dynamic obstacles decreases by 26.30%. This result indicates that the uncertainty introduced by dynamic obstacles affects task success. However, the degradation trend remains relatively moderate. As the proportion of dynamic obstacles increases, the UAVs need to avoid moving obstacles more frequently. Consequently, the AAT and AFD increase slightly by 4.9% and 2.1%. These results indicate that the cooperative strategy based on IER-MADDPG can generate near optimal detour trajectories in dynamic environments. No significant decline in task efficiency is observed, which demonstrates that the proposed algorithm possesses a certain degree of adaptability to dynamic environments.

**Figure 17:**
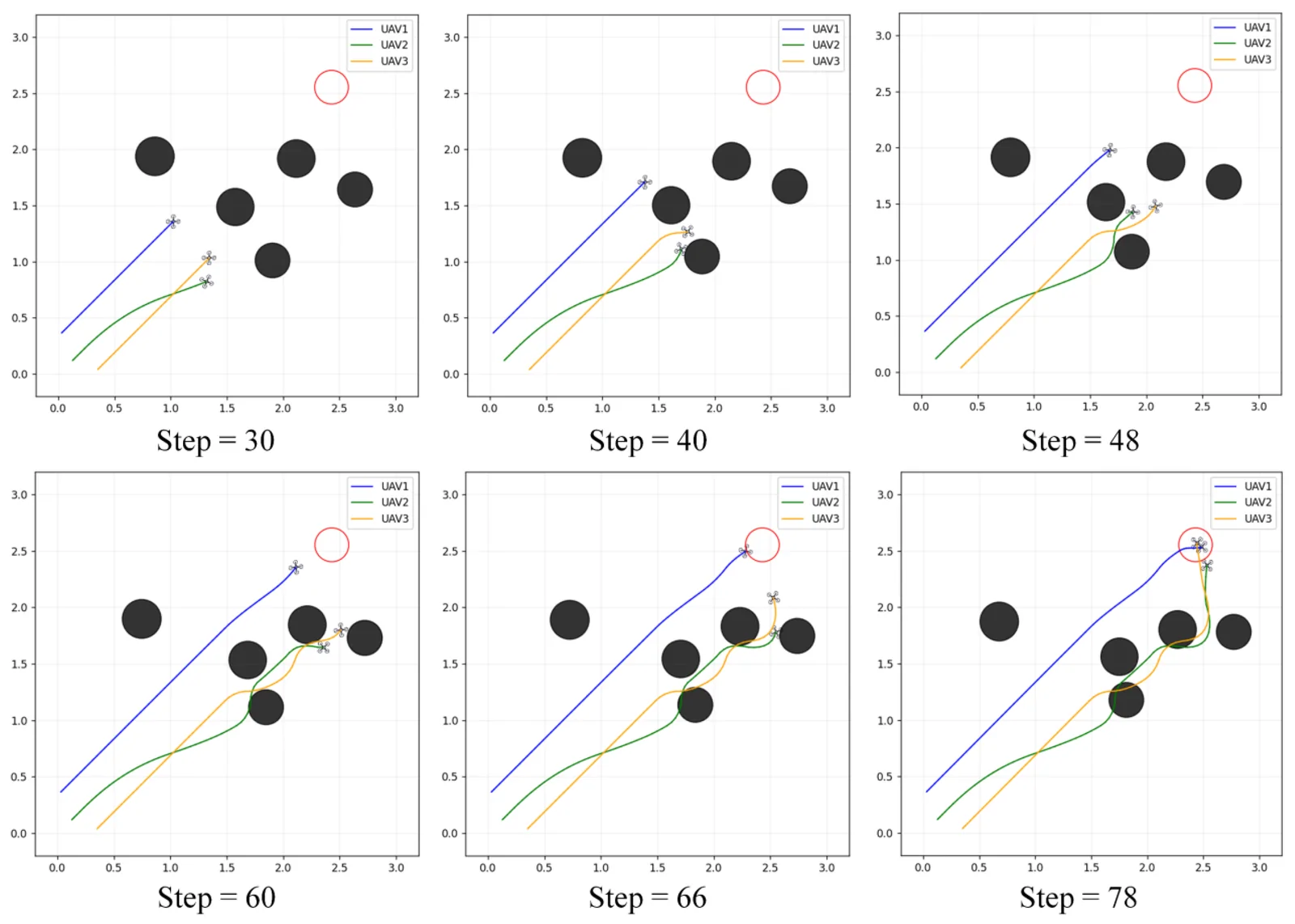
Trajectories of UAV swarm in dynamic-obstacle scenarios.

Figure 17 illustrates the trajectories of the UAV swarm at different time steps in a scenario that contains five dynamic obstacles. The task execution process from Step = 30 to Step = 78 shows the cooperative behavior of the UAV swarm throughout the mission. During the early stage of the test, from Step = 30 to Step = 48, the three UAVs maintain stable safety spacing and clear target orientation. During the middle stage, from Step = 60 to Step = 66, the UAV swarm encounters approaching dynamic obstacles. The UAVs complete obstacle avoidance maneuvers through cooperative adjustments of heading and velocity. No collisions occur during this process. This result verifies the real time cooperative obstacle avoidance capability of the proposed algorithm under dynamic disturbances. During the final stage, at Step = 78, all UAVs safely arrive at the target location, which is represented by the red circle.

## 6. Conclusions

This paper investigates the multi-UAV cooperative path-planning problem in complex obstacle environments and proposes an IER-MADDPG-based cooperative path-planning method. A dual-layer replay buffer structure and a fusion experience sampling mechanism are introduced to improve the utilization efficiency of experience samples and enhance cooperative policy learning. Experimental results demonstrate that the proposed method achieves faster convergence and higher training stability than MADDPG and IL DDPG. In addition, it obtains higher success rates, lower collision rates, and better path-planning performance under different obstacle scenarios. Generalization experiments further verify its effectiveness in environments with different swarm scales and dynamic obstacles. The results indicate that the proposed method exhibits good robustness and adaptability, as well as preliminary scalability within the tested UAV scale, in multi-UAV cooperative path-planning tasks.

Future work will focus on further improving the environmental realism, engineering applicability, and swarm-scale adaptability of the proposed method. First, a more comprehensive three-dimensional UAV kinematic and dynamic model will be established by considering the heading angle, minimum turning radius, aerodynamic constraints, and energy consumption. Second, the current two-dimensional circular-obstacle environment will be extended to more complex scenarios involving irregularly shaped obstacles, three-dimensional terrain, dynamic obstacles, and threat regions with different types and severity levels. Finally, for larger UAV swarms, a hierarchical framework combining task allocation with multi-agent reinforcement learning will be investigated to reduce the coordination complexity and computational burden caused by the increasing number of agents. The performance and scalability of the proposed method will then be further evaluated with a larger number of UAVs and in more realistic environments.

## Figures and Tables

**Table 1:** Hyperparameter settings for algorithm training.

Parameter	Value
Policy network learning rate, $l_{a}$	1 × 10^−4^
Value network learning rate, $l_{c}$	3 × 10^−3^
Replay buffer capacity, $N_{e}$	1 × 10^6^
Batch size, $N_{batch}$	256
Maximum training episodes, E	20,000
Maximum steps per episode, T	110
Discount factor, γ	0.85
Soft update coefficient, τ	0.01
Optimizer	Adam

**Table 2:** Reward function parameter settings.

**Parameter**	**Value**
$\omega_{1}$	4
$\omega_{2}$	0.5
$\omega_{3}$	1
$\omega_{4}$	2

**Table 3:** Performance analysis of cooperative strategies for three UAVs in obstacle scenarios.

Number of Obstacles	Method	SR (%)	CR (%)	AAT	AFD (m)	VC
M=5	IL-DDPG	80.30	19.70	78.71	362.28	0.953
	MADDPG	88.20	11.80	74.55	345.85	0.963
	MATD3	61.05	38.95	78.87	367.00	0.929
	MAPPO	92.60	7.40	84.98	387.67	0.904
	**IER-MADDPG**	**92.55**	**7.45**	**71.70**	**336.04**	**0.971**
M=7	IL-DDPG	60.05	39.95	85.03	373.66	0.947
	MADDPG	80.30	19.70	75.21	351.87	0.959
	MATD3	44.60	55.40	82.01	376.59	0.920
	MAPPO	79.45	20.55	86.97	394.37	0.898
	**IER-MADDPG**	**86.40**	**13.60**	**72.69**	**338.82**	**0.968**
M=9	IL-DDPG	50.55	49.45	85.42	372.97	0.952
	MADDPG	75.00	25.00	75.39	352.65	0.959
	MATD3	41.10	58.90	82.19	376.31	0.918
	MAPPO	79.25	20.75	86.52	394.50	0.898
	**IER-MADDPG**	**85.15**	**14.85**	**73.14**	**340.16**	**0.970**

**Table 4:** Performance metrics with different numbers of UAVs (5 obstacles).

Number of UAVs	SR (%)	CR (%)	AAT	AFD (m)	VC
4	86.90	13.10	71.93	331.61	0.975
5	84.80	15.20	71.82	327.99	0.975
6	73.90	26.10	71.58	325.71	0.976

**Table 5:** Performance metrics in dynamic-obstacle environment (3 UAVs + 5 obstacles).

Obstacle Environment	SR (%)	CR (%)	AAT	AFD (m)	VC
1 Dynamic Obstacle + 4 Static Obstacles	85.60	14.40	72.50	338.42	0.970
2 Dynamic Obstacle + 3 Static Obstacles	79.75	20.25	73.06	338.92	0.969
3 Dynamic Obstacle + 2 Static Obstacles	75.65	24.35	73.30	339.84	0.969
4 Dynamic Obstacle + 1 Static Obstacles	70.70	29.30	75.44	344.49	0.966
5 Dynamic Obstacles	66.25	33.75	76.04	345.53	0.965

## Data Availability

The source code supporting the findings of this study is not publicly available at this stage. It will be made available by the corresponding author upon reasonable request following acceptance of the manuscript. Requests for access to the source code should be addressed to wenlong@cug.edu.cn.
